# Semen Levels of Spermatid-Specific Thioredoxin-3 Correlate with Pregnancy Rates in ART Couples

**DOI:** 10.1371/journal.pone.0061000

**Published:** 2013-05-29

**Authors:** Clayton Buckman, Christophe Ozanon, Jing Qiu, Miriam Sutovsky, Joyce Ann Carafa, Vanesa Y. Rawe, Gaurishankar Manandhar, Antonio Miranda-Vizuete, Peter Sutovsky

**Affiliations:** 1 Division of Animal Sciences, University of Missouri, Columbia, Missouri, United States of America; 2 Departments of Obstetrics, Gynecology & Women's Health, School of Medicine, University of Missouri, Columbia, Missouri, United States of America; 3 Department of Statistics, University of Missouri, Columbia, Missouri, United States of America; 4 Cell and Immunology Core, University of Missouri, Columbia, Missouri, United States of America; 5 Assisted Fertilization Medical Center NATECIA, NOVESCIA Laboratory, Clinique NATECIA, Lyon, France; 6 REPROTEC, Buenos Aires, Argentina; 7 Instituto de Biomedicina de Sevilla, Universidad de Sevilla, Sevilla, Spain; Clermont-Ferrand Univ., France

## Abstract

Spermatid specific thioredoxin-3 (SPTRX3 or TXNDC8) is a testis/male germ line specific member of thioredoxin family that accumulates in the superfluous cytoplasm of defective human spermatozoa. We hypothesized that semen levels of SPTRX3 are reflective of treatment outcome in assisted reproductive therapy (ART) couples treated by *in vitro* fertilization (IVF) or intracytoplasmic sperm injection (ICSI). Relationship between SPTRX3 and treatment outcome was investigated in 239 couples undergoing ART at an infertility clinic. Sperm content of SPTRX3 was evaluated by flow cytometry and epifluorescence microscopy, and correlated with clinical semen analysis parameters, and data on embryo development and pregnancy establishment. High SPTRX3 levels (>15% SPTRX3-positive spermatozoa) were found in 51% of male infertility patients (n = 72), in 20% of men from couples with unexplained, idiopathic infertility (n = 61) and in 14% of men from couples previously diagnosed with female-only infertility (n = 85). Couples with high SPTRX3 produced fewer two-pronuclear zygotes and had a reduced pregnancy rate (19.2% pregnant with >15% SPTRX3-positive spermatozoa vs. 41.2% pregnant with <5% SPTRX3-positive sperm; one-sided p<0.05). The average pregnancy rate of all 239 couples was 25.1%. Live birth rate was 19.2% and lowest average SPTRX3 levels were found in couples that delivered twins. Men with >15% of SPTRX3-positive spermatozoa, a cutoff value established by ROC analysis, had their chance of fathering children by IVF or ICSI reduced by nearly two-thirds. The percentage of SPTRX3-positive spermatozoa had predictive value for pregnancy after ART. Gradient purification and sperm swim-up failed to remove all SPTRX3-positive spermatozoa from semen prepared for ART. In summary, the elevated semen content of SPTRX3 in men from ART couples coincided with reduced incidence of pregnancy by IVF or ICSI, identifying SPTRX3 as a candidate biomarker reflective of ART outcome.

## Introduction

Infertility and the pursuant assisted reproductive therapies (ART) are on rise worldwide. At the same time, oocyte/embryo culture and manipulation technologies, and the diagnostic techniques for female infertility have been advancing steadily. On the contrary, the andrological aspect of infertile couple evaluation still relies mostly on traditional, microscopy based semen analysis, in addition to clinical examinations, hormonal profiles, and cytogenetic and Yq microdeletion profiles. To some extent, the assessment of the DNA fragmentation or chromatin damage, associated with oxidative stress, apoptosis, or male genital tract infection are used as diagnostic tools. Exposure to xenobiotics and advanced paternal age are possible risk factors associated with infertility. Sperm parameters can be measured in specialized laboratories by the DNA-integrity based assays such as SCSA [1], Comet [2] and TUNEL [3], but the value of such exploration is uncertain for the moment [4]. As the repertoire of flow cytometric semen evaluation methods increases, the challenge is on to find new tools and fertility markers to reliably diagnose human male infertility [5]. In addition to DNA fragmentation-based methods, mitochondrial membrane potential probes [6], lectins [7], viability stains [8], apoptosis-detecting probes [9], and antibodies against proteins present predominantly in normal or defective spermatozoa are being adapted for flow cytometric semen analysis (reviewed in [10]).

Thioredoxins (TRX) are a class of redox proteins that function as general protein oxidoreductases by the reversible oxidation of the cysteine residues of their active site whose conserved sequence is Cys-Gly-Pro-Cys. Thioredoxins are maintained in their reduced active form by the flavoprotein thioredoxin reductase (TRXR) at the expense of the reducing power of NADPH [11]. The functions of thioredoxins are typically wide, mostly relying on their redox capabilities, including antioxidant defense, modulation of cell growth and differentiation, regulation of apoptosis and activation of membrane ion channels, among others [11], [12].

Besides ubiquitous thioredoxins found both in cytoplasm and mitochondrial matrix in all cell types, mammals are equipped with an additional set of thioredoxins with an expression pattern restricted to male germ cells [13]. One of such thioredoxins is Spermatid-Specific Thioredoxin 3 [SPTRX3; HUGO nomencalture-TXNDC8 (thioredoxin domain containing 8)]. This thioredoxin is initially observed in the Golgi apparatus of pachytene spermatocytes; it is later retained post-meiotically in round spermatids in close association with the developing acrosomal granule, suggesting a function in acrosomal biogenesis [14]. Once the acrosome is formed, the residual SPTRX3 accumulates in the cytoplasmic lobe of the elongating spermatids ready to be discharged in the form of residual bodies. Interestingly, SPTRX3 remains undetectable in fully differentiated normal spermatozoa of various animal species, but is retained in the superfluous cytoplasm found filling the nuclear vacuoles and wrapping around the sperm tail midpieces in morphologically aberrant human spermatozoa [14], [15]. A dual flow cytometric trial revealed a robust positive correlation between sperm levels of SPTRX3 and ubiquitin (a validated defective sperm biomarker [16]) in a cohort of 19 infertile and 5 fertile subjects [14]. A subsequent study used ImageStream technology combining flow cytometry and light microscopy, to directly demonstrate that the flow cytometric events with high SPTRX3 induced fluorescence are defective spermatozoa with excess cytoplasm and/or nuclear vacuoles [15].

In addition to spermatid specific thioredoxins 1, 2 and 3 [14], [17], [18], [19], the residual cytoplasm carries cytoplasmic enzymes such as glucose-6-phosphate dehydrogenase [20], superoxide dismutase [21], lactic acid dehydrogenase [22] and creatine phosphokinase [20], [23]. Retention of residual cytoplasm coincides with impaired sperm function in humans [24], [25]. In most mammals, the last remnant of spermatid cytoplasm is rejected in the form of a cytoplasmic droplet (CD). Human spermatozoa are unique in that they do not develop a typical CD. The superfluous, redundant cytoplasm (or “excess residual cytoplasm” [26]) in human spermatozoa is an altogether different structure, as it may not be rejected by the spermatozoon in a CD-like manner. It coincides with increased content of reactive oxygen species (ROS) in semen, which at high concentrations are cytotoxic [27]. The ROS generated by excess sperm cytoplasm, or at least by some types of it, can inflict damage by oxidation of unsaturated fatty acids in the sperm plasma membrane, and by DNA breakage, causing reduced motility, pregnancy loss, and embryo morbidity [28].

We conducted the present study to determine the value of the SPTRX3 as a predictor of ART treatment outcome, setting the stage for the validation of SPTRX3 as a biomarker of human male infertility. Our hypothesis was that infertile men will have a significantly higher content of SPTRX3 present in their sperm samples, compared to fertile males. We examined men from 239 couples undergoing infertility treatment because of previously diagnosed female, male, combined (male and female) or idiopathic infertility. We were particularly interested in assessing possible effect of high semen SPTRX3 levels on pregnancy rates.

## Results

### High Flow Cytometric SPTRX3 Values Coincide with Low Pregnancy Rates after ART

**Figure 1:**
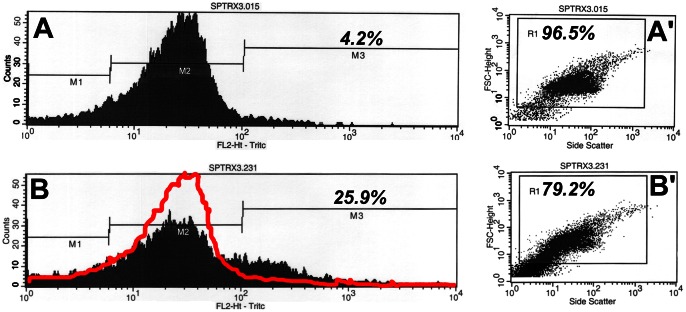
Examples of flow cytometric histograms (A, B) and corresponding light scatter diagrams (A', B') of two male patients with varied SPTRX 3 levels. Histograms, representative of SPTRX3 fluorescene levels in 10,000 cells/sample are divided into marker areas M1 (low fluorescent spermatozoa, sperm fragments and cellular debris), M2 (mostly intact spermatozoa with background-level fluorescence) and M3 (defective, SPTRX3-positive spermatozoa and immature sperm forms with high fluorescence). Percentage of SPTRX3-positive spermatozoa out of total sperm content, including markers M1, M2, and M3 is shown. Gate R1 in scatter diagrams of visible light separates spermatozoa (each dot is one cell) from other flow cytometric events; resultant percentage is percent of high/low fluorescent spermatozoa from total number of flow cytometric events measured per sample in order to acquire 10,000 measurements. Sample B was donated by a patient previously diagnosed with male infertility (oligo-astheno-teratozoospermia; 5 million sperm/ml, 30% motility, 22% normal morphology). Flat histogram-curve and a secondary peak within marker area M3 coincide with 25.9% of SPTRX3–positive spermatozoa in this sample. Sample A was donated by a presumably fertile man with acceptable clinical semen parameters (80 million/ml, 50% motility, 74% normal morphology) whose female partner suffered from endometriosis.

Sperm content of SPTRX3 was evaluated by flow cytometry and correlated with pregnancy rates in 239 couples treated by IVF and/or ICSI. The division of flow cytometric histograms into markers M1-M3 (**Figure** **1**) allowed us to distinguish between cellular debris (M1), cells not carrying detectable SPTRX3, with only a background-level of fluorescence (M2) and SPTRX3-positive, defective spermatozoa (M3). The average % of SPTRX3-positive cells (%M3) for the whole cohort of 239 couples was 14.3%. The average pregnancy rate for all 239 couples enrolled in this study was 25.1%. It was 29.8% for ICSI treated couples (n = 131) and 19.4% for IVF couples (n = 108). Average pregnancy rate for couples above the average %M3 level (n = 83) was 20.5%. Average pregnancy rate for couples below the average %M3 (n = 156) was 27.6%. The median %M3 value, i.e. the value of %M3 at which half the subjects had higher and half had lower SPTRX3 levels, was 12.01%. Average pregnancy rate for couples above the median was 20.2%. Average pregnancy rate for couples below the median was 30.3%, which was significantly higher than the average pregnancy rates of the above-median group (p = 0.04).

**Figure 2:**
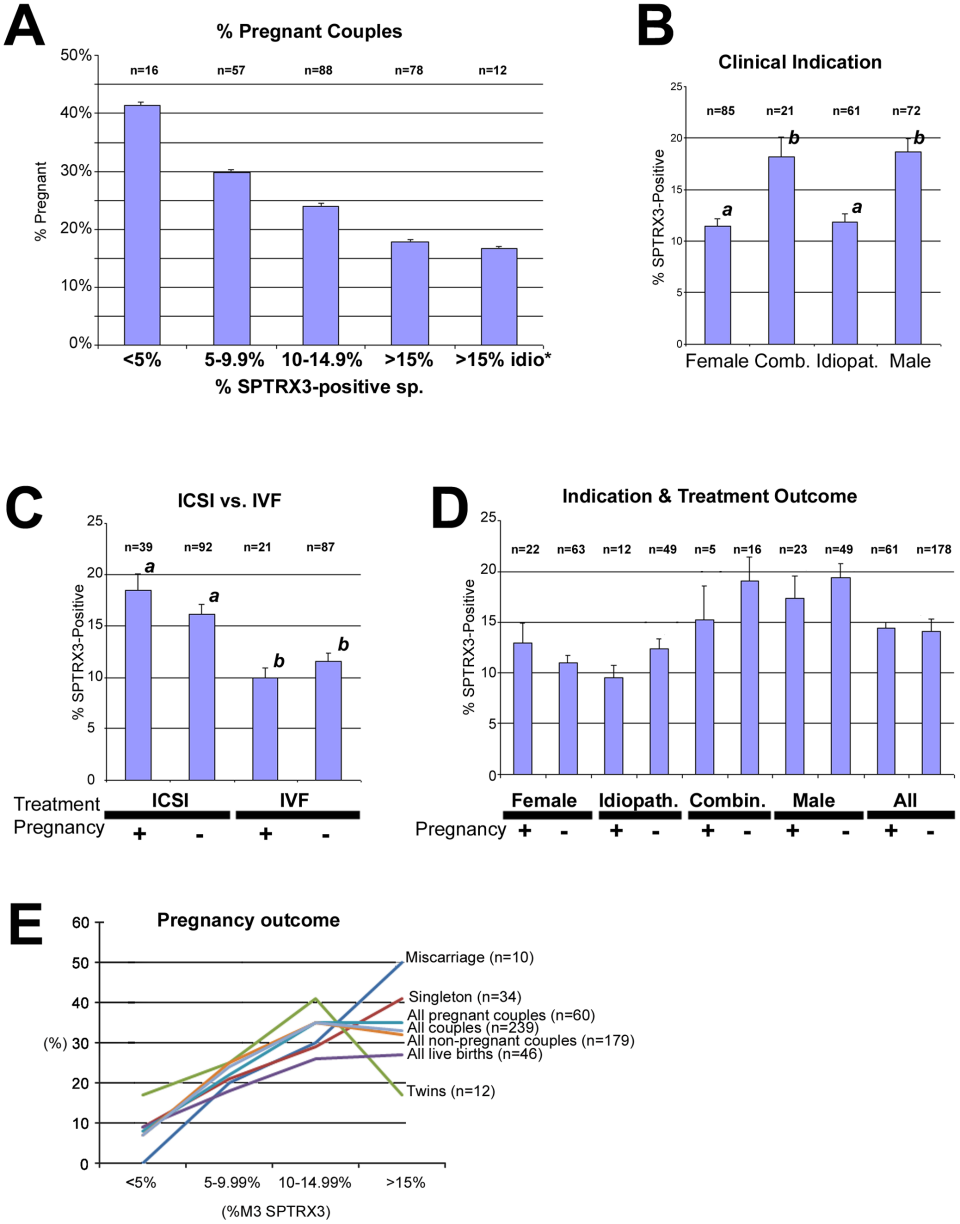
Diagrams of average SPTRX3 content in spermatozoa of men from 239 infertile couples. (**A**) Average pregnancy rates after infertility treatment (IVF or ICSI) decreased gradually with the increased sperm SPTRX3-content in 239 couples divided by % of SPTRX3 spermatozoa (flow cytometric %M3 value; x-axis) into subgroups with less than 5%, 5–9.99%, 10–14.99%, and more than 15% of SPTRX3-positive spermatozoa. Compared to men with less than 5% SPTRX3-positive spermatozoa, those carrying more than 15% of such cells had their chance of conceiving reduced by a half (41.2% pregnant, vs. 19.2% pregnant; one-sided p<0.05). (**B**) Low levels of sperm SPTRX3 were found in female and idiopathic infertility-couples, compared to male I an combined male & female infertility-couples. Different superscripts mark statistically significant differences between columns at p<0.001. The most significant difference was observed between female and male infertility couples (p<0.001). (**C**) Couples treated by IVF had significantly lower average SPTRX3-levels than those treated by ICSI, regardless of whether or not they achieved a pregnancy. Superscripts *^a, b^* mark statistical difference at p<0.05. The most significant difference was found between pregnant IVF couples and pregnant ICSI couples (p<0.001). (**D**) Couples with female and idiopathic infertility had significantly lower SPTRX3 levels (average %M3-values) than couples previously diagnosed with male or combined (male and female) infertility. (**E**) Distribution of SPTRX3 levels within cutoffs in patients divided based on pregnancy outcome. Note that the couples delivering twins had the highest percentage of men with %M3 SPTRX below 15% and loweste percentage of men with >15% M3 SPTRX3.

The dosage effect of SPTRX3 on pregnancy rates was captured when all 239 couples were divided into four groups based on %M3-SPTRX3 values (less than 5% SPTRX3-positive spermatozoa; 5–9.99% positive spermatozoa, 10–14.99% positive spermatozoa; 15% or more SPTRX3-positive spermatozoa). In this partition, the percentage of pregnant couples decreased progressively with the increased SPTRX3 values (**Figure** **2A**). The likelihood of pregnancy was further reduced if only the high SPTRX3 men from idiopathic couples, were analyzed (**Figure** **2A** **;** last column). Average pregnancy rate of this group (n = 12) was only 16.7%. Compared to men with less than 5% SPTRX3-positive spermatozoa, those carrying more than 15% of such cells had their chance of conceiving reduced by a half (41.2% pregnant, vs. 19.2% pregnant; one-sided p<0.05). The ROC analysis revealed that the cutoff values of 12.5% and 15% had a good balance of test sensitivity and specificity (see **Table** **S1 in File S1**). The average pregnancy rate in couples treated by ICSI and having >15%% SPTRX3-positive spermatozoa (n = 60) was 36.7%. Average pregnancy rate was only 11.1% in IVF couples with >15% positive spermatozoa (n = 18). The IVF couples with <15% SPTRX3-positive spermatozoa (n = 90) achieved a pregnancy rate of 21.1%.

Discriminant analysis revealed that %M2 SPTRX3 was a significant positive predictor of pregnancy in IVF couples and the median M3 was a significant negative predictor for the ICSI group (see **Supplemental Statistics – Section ANALYSIS I & Table** **S11 in File S1**). Based on clinical indication, SPTRX3 levels (%M3) were significantly higher (p<0.05) in couples with male and combined (male and female) infertility, compared to couples with female-only or idiopathic infertility (**Figure** **2B**). Also, SPTRX3 levels were significantly higher (p<0.001) in ICSI couples compared to IVF couples (**Figure** **2C**). Within the ICSI-treated group, couples that achieved pregnancy had numerically higher %SPTRX3 positive spermatozoa, but this increase was not statistically significant. The levels of sperm SPTRX3 were not significantly different between smokers and non-smokers when all couples were evaluated (**Figure** **S2**). Pregnancy outcomes were confirmed in 57/60 pregnant couples; 46 of those couples delivered babies (confirmed live birth rate of 19.2%)(**Table** **S2A in File S1**). Lowest average SPTRX3 levels were found in couples that delivered twins (n = 12; **Figure** **2E** **; Table** **S2B in File S1**), compared to those delivering a singleton (n = 34) and those that miscarried (n = 10). In addition to pregnancy and live birth rates, parameters of embryo development and numbers of oocytes and embryos produced per couple were analyzed. Numerically, couples in which men had less than 5% of SPTRX3 positive spermatozoa produced most normal two-pronuclear zygotes and transferable embryos (**Table** **S3 in File S1**). However, no significant correlations were found between parameters of early embryo development and flow cytometric SPTRX3 levels.

### Flow Cytometric SPTRX3 Levels Reflect Clinicians' Treatment Decision, and Reveal Undiagnosed Male Infertility in Idiopathic Infertility Cases

The SPTRX3 levels in all 239 couples were compared based on pregnancy outcome and clinical indication (**Figure** **2D**). Compared to female infertility couples, the average %M3 value of SPTRX3 in diagnosed male infertility cases was increased by 63%. Elevated SPTRX3 levels (>15% M3) coincided with the clinician's decision to treat by ICSI. Consequently, logistic regression and discriminant function analysis were used to determine the relationship between treatment assignment (ICSI or IVF) and semen SPTRX3 levels (**Table** **S4 in File S1; Supplemental Statistics**, section **ANALYSIS II & Table** **S12, S13 in File S1**). Among 16 flow-cytometric parameters, only median M2, Mean M2 and %M1 were not significant for predicting the treatment assignment. Mean M1 and Median M1 had the largest influence on the odds of being assigned to IVF treatment. The odds of being assigned to IVF treatment increased by over 34 fold and 4 fold for every unit of increase in Mean M1 and Median M1, respectively. However, we noticed that the ranges of Mean M1 and Median M1 were small (their values varied between 1 and 4). The next three most influential predictors were CV M1, %M2 and %M3. For every unit increase in CV M1 and %M3, the odds of being assigned to FIV treatment decreased by 16.3% and 9.6%, respectively. For every unit increases in %M2, the odds increased by 14.1%. Discriminant analysis identified %M2 SPTRX3 as a positive discriminant for IVF assignment and Mean M3, Median M3 and % M3 SPTRX3 as positive discriminants for ICSI assignment. Thus, increased SPTRX3 content in spermatozoa can be used as an indication for ICSI treatment. Among the six conventional semen parameters, only sperm volume was not significant predictor for treatment assignment (**Table** **S4 in File S1**). Motility parameters, PR and M gave the most explanatory powers with odds ratio of 1.176 and 1.142 (i.e. the odds of being assigned to IFV treatment increases 17.6% and 14.2% for every unit increase in PR and Mob measurements, respectively).

### Flow Cytometric SPTRX3 Values Reflect Sperm SPTRX3 Retention Phenotypes Observed by Epifluorescence Microscopy

**Figure 3:**
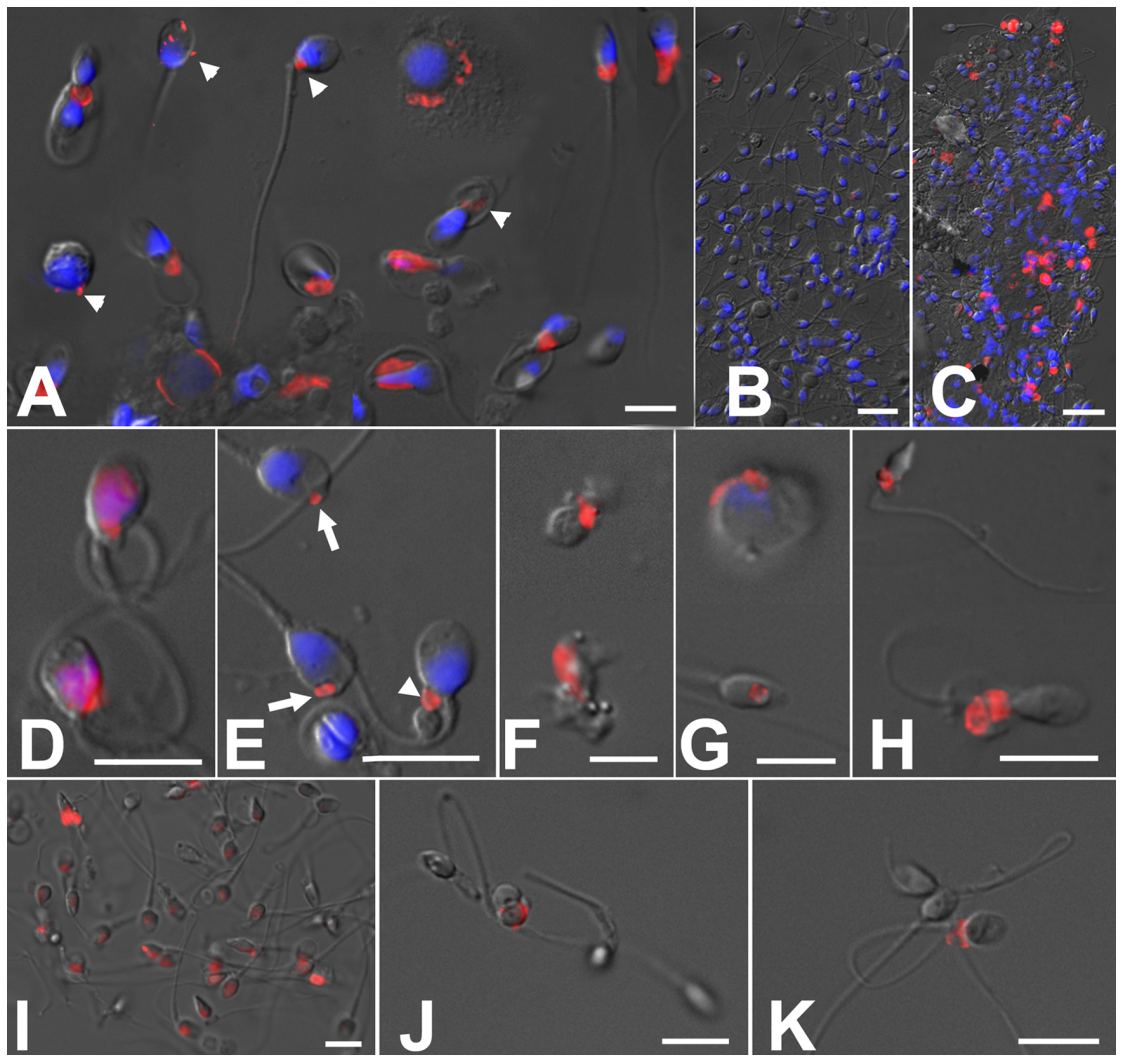
Epifluorescence microscopy of sperm samples processed for flow cytometric measurements of SPTRX3 (red). The DNA-stain DAPI (blue) was added after flow cytometric analysis. Epifluorescence images are superimposed onto parfocal differential interference contrast (DIC) images (gray). (**A**) Examples of SPTRX3 labeling in samples from male infertility patients. Arrowheads point to minor carryovers of residual cytoplasm in spermatozoa and immature sperm forms; some of these cytoplasmic structures may not be detectable by conventional transmitted light microscopy, but can be identified accurately by SPTRX3 labeling. (**B, C**) The difference in SPTRX3 content is obvious even at low magnification between a sample from a presumed fertile donor (**B**) and the one of a patient previously diagnosed with oligo-astheno-teratozoospermia (**C**). (**D–G**) High magnification renderings of various patterns of SPTRX3 retention in defective spermatozoa, including a predominant sperm head/nuclear labeling (**D**), minor SPTRX3 retention in the sperm head (**E;** arrows) and sperm tail connecting piece (**E;** arrowhead), sperm-derived cellular debris free of DNA/chromatin (**F**), immature, spermatid-like form (**G**; top), and SPTRX3-filled large nuclear vacuole (**G**; bottom). (**H**) Immunolabeling of SPTRX3 in a frozen-thawed semen sample labeled according to a simplified protocol omitting sperm fixation and washing. (**I–K**) Detection of SPTRX3 before (**I**) and after semen purification on PureSperm gradient (**J**), and in a sample purified by swim-up after PureSperm treatment (**K**). Defective spermatozoa are not removed completely by this two-step procedure. Scale bars are 10 µm in all panels except B, C, where they represent 20 µm.

Immunofluorescence processing and subsequent analysis of a subset of 150 samples by epifluorescence microscopy identified varied phenotypes of SPTRX3- retention by defective spermatozoa, ranging from spermatozoa bearing a small dot of SPTRX3 (**Figure** **3A, 3E**) to the highly SPTRX3-positive spermatozoa (**Figure** **3D, 3G** **)** and SPTRX3-containing cellular debris (**Figure** **3F**), readily obvious even at low magnification (compare **Figure** **3C** to **F** **igure** **3B**). Similar labeling was obtained in spermatozoa immunolabeled without fixation (**Figure** **3H**). Some SPTRX3 positive spermatozoa persisted in semen samples purified by gradient centrifugation and swim-up (**Figure** **3** **I–K**). Non-sperm elements/bodies present in the analyzed samples did not display detectable SPTRX3 fluorescence. The epifluorescence microscopy-analysis of SPTRX3 correlated with flow cytometric evaluation of this subset of 150 patients and was reflective of pregnancy rates (**Figure** **S3 in File S1;** **Table** **S5A, S5B in File S1**).

### Effect of High SPTRX3 Content on Pregnancy Rates after Adjustment for Partners' Age

Female patients above 35 years of age tend to produce fewer oocytes after superovulation, and these oocytes are perceived to be of lesser quality and developmental potential. It was thus useful to assess whether high semen content of SPTRX3 in male partners of female patients 35 years or older could further aggravate couples' infertility. When the couples were divided based on female age and male content of SPTRX3 (**Table** **S6 in File S1**), and by age and indication (**Table** **S7 in File S1**), there was a non-significant trend towards higher pregnancy rates in low SPTRX3 couples regardless of female age. Male age itself did not show correlation with pregnancy rates, SPTRX3 levels or conventional semen parameters. There was a moderate-to-high correlation between flow cytometric data and several parameters of conventional, clinical semen analysis (**Table** **S8 in File S1**).

Next, the logistic regression analysis was applied to test the relationship of pregnancy rate with the sperm quality parameters, taking into consideration of female age and treatment assignments (**Supplemental Statistics – ANALYSIS III in File S1**). Considering only the patients assigned to IVF treatment, both %M2 and %M3 SPTRX3 were significant predictors of pregnancy rate, with or without adjustment for the female age factor. Importantly, none of the conventional semen parameters, or other remaining 14 flow cytometric SPTRX3 parameters, was significant even with adjustment for the female age. The odds of getting pregnant in the IVF group were significantly reduced by increased %M3 value, with (p<0.05) or without (p<0.05) adjustment for maternal age. The %M2 was also a significant predictor for pregnancy rate for IVF group (p<0.02/p<0.05 with/without age adjustment).

For the ICSI group, the pregnancy rate was not affected by any of the flow cytometric or conventional sperm quality parameters or the female age factors. This is not surprising since ICSI can overcome a wide range of sperm anomalies that would hinder conventional IVF treatment. When considering all patients together, %M2 and %M3 SPTRX3 were not significant predictors for pregnancy rate (p>0.1). However, when we categorized the %M3 levels into four groups (below 5%, between 5–10%, between 10–15%, above 15%), the estimated odds ratio between the below 5% group and the above 15% group had a p-value = 0.06. After adjusting for female age above/below 35 years, the adjusted odds ratio for the <5% SPTRX3 group over the >15% SPTRX3 group had a p<0.05. Using 85% as a cutoff for %M2 (normal spermatozoa), the below 85% group and above 85% group had significantly different pregnancy rates when considering all patients together. The odds of getting pregnant for the >85% %M2 group were significantly higher than that for the <85% group (p<0.05) and the pregnancy rate was 41.6% versus 22.72%. After the adjustment for the female age above 35, the odds ratio remained significant (p<0.05).

### Relationship between Flow Cytometric SPTRX3 Parameters, Clinical Semen Parameters and Clinical Diagnosis of Male Infertility

This analysis was performed using logistic regression analysis *(SAS proc log)* of SPTRX3 parameters and conventional semen parameters including sperm concentration (C), semen volume (V), % normal spermatozoa (NX), progressive motility (PR), % motile (M) and % necrotic (NEC)(**Table** **1**). All parameters except V were significant predictors for the clinical diagnosis of male infertility (**Table** **2**). NX, PR, M and C were negative predictors for male infertility. Among 16 flow cytometric parameters (**Table** **2** **)**, Mean M1, Median M1, CV M1, %M2 and %M3 were the most sensitive predictors for male infertility. Discriminant function analysis identified PR, M, NX, C, %Total and %M2 SPTRX3 as the discriminants correlated positively with sperm quality (**Supplemental Statistics –** Section **ANALYSIS IV, Table** **S14, S15 in File S1**). Negative discriminants included %M3, Mean M3 and Median M3 SPTRX3. Analysis based on etiology/testiculopathy (**Table** **3**) revealed highest levels of SPTRX3 in men from male infertility couples (n = 72) diagnosed with oligo-asthenozoospermia. Couples with astheno-teratospermia had the lowest SPTRX3 and highest pregnancy rates. Altogether, 97.2% of male-only infertility couples were treated by ICSI. Spearman rank correlation was used to confirm the relationship between sperm SPTRX3 values and conventional semen parameters. Five most informative flow cytometric SPTRX3 parameters (Mean M1, Median M1, CV M1, %M2, %M3) were significantly correlated with conventional semen parameters, particularly with motility parameters M and PR (**Table** **S9 in File S1**). The %M3 parameter, capturing cells with high SPTRX3 content, also correlated negatively with % normal spermatozoa (NX) and sperm count (C), and positively with % necrotic spermatozoa (NEC).

**Table 1:** Average clinical semen parameters in 239 male patients divided by %M3 SPTRX3 and pregnancy.

%M3 SPTRX3	N	% pregnant		Semen volume	Sperm concentration	% dead spermatozoa	% motile spermatozoa	% progressively motile spermatozoa	% normal spermatozoa
**<5%**	16	**41.2%**	Mean	**2.4**	**95.2**	**22**	**49**	**28**	**48**
			SE	0.3	21.3	2	2	3	4
**5–9.99%**	57	**29.8%**	Mean	**3.2**	**83.6**	**27**	**46**	**27**	**46**
			SE	0.2	7.1	1	1	1	2
**10–14.99%**	88	**23.9%**	Mean	**3.3**	**71.1**	**28**	**42**	**24**	**42**
			SE	0.2	5.0	1	1	1	2
**>15%**	78	**19.2%**	Mean	**3.3**	**40.6**	**33**	**33**	**16**	**35**
			SE	0.2	5.2	2	2	1	2
**>15% idiopathic**	12	**16.7%**	Mean	**3.2**	**64.0**	**30**	**44**	**24**	**45**
			SE	0.3	18.4	4	2	2	4
**Pregnant**	60	100%	Mean	3.2	65.8	29	38	20	38
			SE	0.2	7.5	1	2	1	2
**Non-pregnant**	179	0%	Mean	3.2	65.8	29	41	23	43
			SE	0.1	4.0	1	1	1	1

The only group that shows somewhat reduced average clinical semen parameters are the men with highest SPTRX3 levels (>15% M3). Idiopathic infertility patients with apparently high SPTRX3 level (>15% idiopathic; bottom row) shows acceptable, normal clinical semen parameters. David's classification scheme for sperm morphology was employed [32], [33]. SE = standard error.

**Table 2:** Relationship between the clinically diagnosed male infertility and individual, conventional and flow cytometric SPTRX3 parameters reflective of sperm quality.

Parameter	p-value	Odds ratio*
V	0.9833	NA
PR	<0.0001	0.677
NX	<0.0001	0.903
NEC	<0.0001	1.07
M	<0.0001	0.772
C	<0.0001	0.948
Median ALL	0.0007	1.055
Median M3	<0.0001	1.028
Median M2	0.0176	1.074
Median M1	<0.0001	0.08
Mean all	<0.0001	1.055
Mean M3	<0.0001	1.022
Mean M2	0.0249	1.084
Mean M1	<0.0001	0.014
Total	<0.0001	0.915
%M3	<0.0001	1.12
%M2	<0.0001	0.869
%M1	0.8212	NA
CVALL	0.0005	1.007
CVM3	0.0131	1.012
CVM2	0.3455	NA
CVM1	<0.0001	1.241

An odds ratio smaller than 1 indicates that the parameter is negatively associated with the odds of being diagnosed with male infertility. A larger than 1 odds ratio indicates a positive relationship between the parameter and the clinical diagnosis of male infertility. An odds ratio larger than 1, e.g. 1.12 is interpreted as “the odds of being diagnosed with male infertility increase by 12% for every unit of increase in the parameter measurements.” An odds ratio smaller than 1, e.g., 0.014, is interpreted as “the odds of being diagnosed with male infertility decrease by 98.6% for every unit of increase in the measured parameter.” Abbreviations: C = sperm count; V = semen volume; M = total motility; PR = progressive motility; NX = morphology/% normal spermatozoa; NEC = percent of necrotic spermatozoa assessed by Williams test.

**Table 3:** SPTRX3 levels and ART outcomes in 72 male infertility patients divided by etiology.

Aethiology	A	AT	OA	OAT	AZ*	Other**	AVG/Total
Mean %M3 SPTRX3	18.4	14.8	28.2	18.4	25.6	17.6	18.7
SE	2.6	2.5	7.2	1.6	8.4	3.9	1.2
N	14	9	4	33	3	9	72
Pregnant/total (%)	2/14 (14%)	4/9 (44%)	0/4 (0%)	12/33 (36%)	3/3 (100%)	2/9 (22%)	23/72 (32%)
ICSI-treated/total (%)	12/14 (86%)	9/9 *** (100%)	4/4 (100%)	33/33 (100%)	3/3 (100%)	9/9 (100%)	70/72 (97%)

Most frequent etiologies included asthenozoospermia (A), astheno-teratozoospermia (AT), oligo-asthenozoospermia (OA), and oligo-astheno-teratozoospermia (OAT). One patient had obstructive azoospermia and one had obstructive oligo-teratozoospermia. Other, less frequent etiologies included teratozoospermia (2 patients), obstructive (1), vasectomy (1), varicocele (1), anti-sperm antibodies (2) and asthenozoospermia with anti-sperm antibodies (1). One couple reported failed IVF before being treated by ICSI. Couples diagnosed with combined male & female, female-only or idiopathic infertility excluded from this analysis/table.

A = asthenozoospermia.

AT = astheno-teratozoospermia.

OA = oligo-asthenozoospermia.

OAT = oligo-astheno-teratozoospermia.

* Obstructive.

** Other  =  teratozoospermia (2 patients), oligo-teratozoospermia, obstructive (1), vasectomy (1), varicocele (1), anti-sperm antibodies (2), asthenozoospermia with anti-sperm antibodies (1).

*** One couple reported failed IVF before being treated by ICSI.

## Discussion

Present statistical analyses indicate that high semen content of SPTRX3 in infertile men translates into a reduced chance of impregnating their female partners via ART. In fact, couples' pregnancy rates were significantly lower for men with >15% SPTRX3 sperm compared to men with <5% SPTRX3 cells. Since the treatment decision/assignment in the examined 239 cases was in part based on semen analysis, it is no surprise that men from IVF couples had significantly lower average SPTRX3 levels than men from couples that were treated by ICSI. That the increased levels of SPTRX3-positive spermatozoa predispose couples for ICSI treatment is obvious in spite of the fact that several couples were initially treated by IVF, without success, before the ICSI treatment was applied. Altogether, pregnancy rates fell progressively with increasing semen SPTRX3 levels.

When broken down into ICSI and IVF groups, couples divided by %M3 SPTRX3 showed high pregnancy rates in ICSI group with <5 %M3. On average, ICSI and male infertility cases had significantly higher %M3 values but also slightly higher pregnancy rates than IVF and female-only infertility cases. This is understandable because most of these cases were oligo-astheno-teratozoospermic and therefore treated by ICSI. The IVF patients had overwhelmingly female-only infertility, with poor oocyte quality being the likely contributor to a high incidence of failed IVF treatment (75%). However, this could also have been compounded by undiagnosed male infertility, indicated by high SPTRX3 levels in 20% of those presumed female-only infertility couples. Consequently, the percentage of mature oocytes that formed two pronuclei after assisted fertilization decreased in a stepwise manner, in parallel with increasing %M3 SPTRX3 in both the IVF-treated and ICSI-treated couples. Altogether, high sperm SPTRX3 levels seem to coincide with clinical decision to treat by ICSI. The SPTRX3-levels corroborate previous clinical diagnosis of male infertility, and reveal undiagnosed male infertility in a fraction of cases with idiopathic and female-only infertility.

While the majority of the 239 couples in this study were assigned to IVF or ICSI based on their clinical test results, our SPTRX3 results indicate that approximately 20% of IVF couples would have profited from ICSI because of undiagnosed male infertility. **Table** **S3 in File S1** shows that sperm SPTRX3-levels but not couples' clinical diagnosis, in part based on subjective semen analysis, coincided with successful fertilization and formation of two normal pronuclei in the resultant zygotes. While couples with <5 %M3 values of SPTRX3 produced 2-PN zygotes in 86.2% of fertilized oocytes, idiopathic couples with >15 %M3 did so in only 69.7% of oocytes (see **Table** **S3A in File S1**; column 9). No such differences were observed based on clinical diagnosis/indication (**Table** **S3B in File S1**). There was no significant difference in %M3 values of SPTRX3 between female and idiopathic cases. However, 20% of the idiopathic cases showed high SPTRX3 values (>15% M3), indicating that these were in fact male infertility cases. Similarly, 20% of presumed fertile males in couples diagnosed with female-only infertility also had >15% SPTRX3-positive spermatozoa. Together with the effect of SPTRX3 on pregnancy rates, these data illustrate the predictive value of SPTRX3-based semen analysis for treatment outcome.

**Table** **2** and **Table** **S8 in File S1** reveal relationship between clinically diagnosed male infertility and SPTRX3 parameters. Furthermore, **Table** **1** shows that men with high SPTRX3 levels show acceptable clinical semen parameters, but low pregnancy rates are achieved by their female partners. As shown in **Table** **S8 in File S1**, the levels of correlation (r-values) between flow cytometric parameters (%M3; %TOTAL, GeoMean M3) and conventional semen parameters are varied, depending on how the patient pool was subdivided based on indication, treatment decision and treatment outcome. All three flow cytometric parameters show median level of correlation with some clinical semen parameters in some subgroup but not in others. This indicates that sperm SPTRX3 values are relatively independent of conventional semen parameters. The flow cytometric parameter most closely correlated with clinical semen parameters is the %TOTAL, which reflects the percentage of flow cytometer-registered events corresponding to intact spermatozoa (both morphologically normal and abnormal) out of all objects passing through a flow cytometer, including sperm fragments and debris present in semen sample. This is based on visible light scatter of registered events rather than on SPTRX3-induced fluorescence. The proposition of a sperm quality test that is relatively independent of conventional semen parameters but predictive of pregnancy outcome arrives at a time of renewed calls for more accurate semen analysis. Some recent studies even go as far as implying that even the strictest, most accurate morphological criteria could lead to diagnosis of teratozoospermia that forces unnecessary ICSI treatment in couples treatable by IVF [29].

No single biomarker is a universal indicator of male infertility and treatment outcome; however, the SPTRX3-based assay could be combined with other immunocytochemical and cytochemical tests such as the ubiquitin-based sperm quality assay [16] or a test of sperm acrosomal status based on lectin labeling [7]. SPTRX3 is a unique marker because it is a well characterized, germline-specific protein with a distinct localization pattern in spermatozoa, and there is no need to measure its relative abundance in individual cells to decide which cells are “positive” and which are “negative.” Most normal spermatozoa do not contain amounts of SPTRX3 detectable by immunofluorescence. Simply counting positive and negative spermatozoa is straight-forward and, if desired, such a test gives numbers that could be used to set up clear cutoffs for normal and infertile males. However, thresholds will be different for manual, epifluorescence microscopy based assessments as opposed to automated flow cytometric measurement. Discriminant function analysis of flow cytometric data and clinical diagnosis showed that the mean % of SPTRX3-postitive spermatozoa (%M3) was for 11.6% in presumed fertile men vs. 18.6% in the clinically diagnosed infertile men (**Supplemental Statistics – ANALYSIS IV in File S1**). The cutoff values of 12.5 and 15% were found to have a good balance of sensitivity and specificity in ROC analysis (**Table** **S1 in File S1**). A 10% cutoff value had sensitivity close to 90%, but this was at the expense of specificity. The threshold/cutoff levels for an infertile male could also differ between IVF patients (e.g. 7.5 or 10% positive) and ICSI patients (e.g.15 or 17.5%). Thus, SPTRX3-based test could be used as a decision making tool when physicians have to decide whether to use intrauterine insemination, IVF, or ICSI. For example, a treatment with intrauterine insemination could be used in cases when a male partner's SPTRX3 levels are below 10% positive spermatozoa, IVF for men with between 10–15% SPTRX3 positive spermatozoa, and ICSI for men with >15% positive spermatozoa.

Similar to flow cytometric SPTRX3 analysis, the simplified, light microscopic analysis revealed higher pregnancy rates in couples with low SPTRX3 levels (**Table** **S6 in File S1**). While the more sensitive flow cytometric analysis would be preferable, the simplified light microscopic test could become a valuable tool for clinical semen evaluation wherever flow cytometry is not convenient or available. This could be done either by epifluorescence microscopy (direct conjugation of a fluorescent probe to anti-SPTRX3 antibody), or by simple transmitted light microscopy using anti-SPTRX3 antibody conjugated to horse radish peroxidase and developed by applying alkaline phosphatase or DAB. To further facilitate sample staining, sperm spreads would be fixed by methanol and dried directly on the microscopy slides. Even so, development of simplified protocols for flow cytometric semen analysis is necessary. As shown in Fig. 3 H, spermatozoa can be labeled with antibodies without previous fixation, i.e. by a mild permebilization and immunolabeling. Permebilization kills spermatozoa but in this case, it cannot be omitted because the SPTRX3 antigen is concealed under the plasma membrane of the membrane-intact spermatozoa. This protocol will address the major disadvantage of SPTRX3 test compared to other flow cytometry based sperm test such as SCSI or TUNEL, i.e. the lengthy, multi-step processing. This approach would benefit the new generation of dedicated sperm analysis flow cytometers such as Easy Cyte Plus, a capillary flow-based device with pre-set sperm tests, used to analyze small volumes of sperm suspensions [30]. The SPTRX3 based test may be a valuable addition to the aforementioned flow cytometric tests. Out of five male infertility patients tested by SCSI in the present cohort, none had the DNA fragmentation index (DFI) above the threshold level of 20% (Mean DFI = 7.8±2.2%; range 2–14%). However, the average %M3 SPTRX3 for these five patients was 25.1±7.0% (range 7.8-50.0%), compared to 18.7±1.2 %M3 of the entire cohort of male infertility patients. Future trials will be designed to examine the relationship between SPTRX3 retention and aberrant sperm chromatin structure.

The SPTRX3-positive spermatozoa most likely correspond to spermatozoa commonly referred to as “immature sperm” [31]. Superfluous cytoplasm can be very conspicuous in some of these spermatozoa, but difficult to detect by conventional light microscopy in others, even with high magnification lenses and DIC contrast optics. The use of SPTRX3 as a biomarker could allow clinicians to identify defective spermatozoa during semen analysis that would be undetectable to conventional semen evaluation. The SPTRX3 based method is robust, and as demonstrated here, capable of detecting sperm defects in men from couples diagnosed with idiopathic infertility. Altogether, the present study documents the relationship between SPTRX3 levels and pregnancy rates after ART, and the effect of SPTRX3 on quality of zygotes (number of pronuclei); it also identified differences in semen content of SPTRX3 and phenotypes of its retention between male patients from couples treated by ICSI and those treated by conventional IVF. Consequently, it may be possible, and necessary to set the threshold levels for normal/elevated semen SPTRX3 content differently for these two cohorts of infertility patients. Altogether, SPTRX3 protein is a candidate biomarker of sperm dysfunction that is relatively independent of clinical semen parameters, and relevant to decision making in ART.

## Materials and Methods

### Semen Samples and Study Design

Semen samples were donated by men from 239 consenting couples, free of HIV and any other detectable contagious diseases, presenting for infertility treatment at the Assisted Fertilization Medical Center, Clinique Monplaisir, Lyon, France. Semen was collected on the day of IVF/ICSI and the leftover was sample cryopreserved within two after collection hours. Identities of donors were not disclosed to researchers involved in this study. All couples also consented to sharing of diagnostic data and treatment outcome-data. Couples were classified and assigned as follows: Female infertility included hydrosalpinx, endometriosis and polycystic ovary syndrome (PCOS); these couples were assigned to IVF since their sperm parameters were within acceptable limits by WHO criteria. This group also included ovarian insufficiency revealed by ultrasonographic baseline antral follicle count, associated with other pathologies; such cases were treated by ICSI. Male and combined male and female infertility was diagnosed based on semen analysis, as described below. Clinical indications of male infertility, as defined by WHO, included asthenozoospermia (A), astheno-teratozoospermia (AT), oligo-asthenozoospermia (OA) and oligo-astheno-teratozoospermia (OAT). Cases of obstructive azoospermia (AZ), obstructive OAT, teratozoospermia, obstructive OT, azoospermia due to vasectomy, varicocele, and anti-sperm antibodies were present. In vasectomy cases and cases of obstructive OAT due to inflammation, spermatozoa for ICSI were collected from testis. Five male infertility patients were tested for chromatin structure/DNA fragmentation by SCSI at a commercial laboratory, but all five had acceptable DNA-fragmentation index (<20% DFI). Generally, IVF was used in cases with acceptable progressive motility, while cases of OAT were treated by ICSI. Idiopathic infertility (no clinical symptoms or pathologies, and no previous history of andrological problems in male partners) were treated by ICSI if they reported previous failures with intrauterine insemination (IUI). If there was no history of IUI, couples were assigned to IVF treatment. In several cases, couples were assigned to ICSI treatment because of reported IVF failure.

Male infertility was diagnosed by applying conventional WHO cutoff values valid at the time of sample collection (2002–2003) for sperm count (C), semen volume (V), total motility (M), progressive motility (PR). Percentage of spermatozoa with normal morphology (NX; % normal spermatozoa) was established by using David's classification [32], [33], which was the most common method for sperm morphology assessment at the time of treatment in France, where all samples and clinical parameters for this study were collected. Volume was measured with a calibrated 15 ml Falcon tube. Spermatozoa were counted using a Thoma Chamber at a lens magnification of 10 or 20×; spermatozoa were immobilized using 36% formaldehyde and stained with methylene blue. Sperm morphology was assessed by Harris and Shorr staining. Percent of necrotic spermatozoa (NEC) was assessed by Williams test (eosin staining). Clinical data were provided to researcher after the completion of flow cytometric data collection. Couples that had ICSI following a failed IVF treatment are included in the ICSI-group for the purpose of analysis. Conventional semen evaluations and cryopreservation were performed at Clinique Monplaisir, Lyon, France. Analyzed samples were cryopreserved in sperm freezing medium (Medicult Origio), in which they were diluted progressively to 1∶1 v/v ratio during a ten-minute incubation at 20°C. Slow cooling from 20°C to −40°C at 2°C/minute was followed by plunging in liquid nitrogen. Cryopreserved sperm samples were processed and analyzed by flow cytometry and epifluorescence microscopy at the University of Missouri, under an approved Internal Review Board (IRB) protocol.

All 239 samples were analyzed by flow cytometry. A randomly chosen subset of 150 samples was examined by epifluorescence microscopy to assess the percentage of SPTRX-3 positive cells (100 spermatozoa examined randomly per slide), and the data were correlated with the flow cytometric data. Both SPTRX3 assays were done on semen samples left over from ART treatment. Western blotting trials (**Figure** **S1 in File S1**) were conducted on three samples from the patients diagnosed with extreme male infertility (dysplasia of the fibrous sheath) and on three samples from presumed fertile donors, purchased from the Fairfax Cryobank, Fairfax, VA. These trials were designed to confirm the specificity of the SPTRX3 antibody used for flow cytometry, and to explore a possibility of SPTRX3 quantification based on densitometry technique.

### ART Treatment, and Evaluation of Embryos and Pregnancy Rates

Spermatozoa for both IVF and ICSI were purified on a 90%/70% PureSperm density gradient by centrifugation at 250×g, diluted and washed at 1,350×g with IVF medium (Medicult, Origio France, Limonest, FR). Sperm pellets were resuspended in 300 µl of IVF medium for IVF or in 100 µl for ICSI. Spermatozoa were incubated for 1 hour at 37°C and 5.5% CO_2_ before ART treatment. Fertilization was performed with 100,000 spermatozoa (125,000/ml) and a maximum of 4 oocytes. *In vitro* fertilization was performed in IVF Medium (MediCult, Origio France) supplemented with sodium pyruvate and synthetic medium replacement (SSR; Origio France). A female patient was considered pregnant if her hCG was above 1000 UI and a gestational sac was detected by ultrasonography. Spermatozoa for ICSI were immobilized in clinical grade PVP medium with HEPES, sodium pyruvate and SSR. Individual spermatozoa for ICSI were selected at a 1,000x magnification based on morphology (David's classification; [32], [33]). In several teratospermic patients (severe oligo-astheno-teratozoospermia), it was impossible to find morphologically normal spermatozoa. The embryos were cultured in Global medium (LifeGlobal; lifeglobal.com). Embryos were evaluated on day 2, 48 hrs post oocyte pick-up, applying the following criteria of normal morphology: Minimum of 4 cells and maximum of 6 on day 2, with regular blastomere size and homogeneous cytoplasm, with <10 % of exudate (cytoplasmic fragments). Only embryos that met the above criteria were used for cryopreservation. Embryos were cultured *in vitro* for up to 6 days (blastocyst stage). The embryos which were not transferred or cryo-preserved on day 2 were re-evaluated on day 6, but the results of the second cryopreservation were not included in this study.

### Sperm Processing for Flow Cytometry

The sperm samples were stored and shipped in individual straws in liquid nitrogen, and processed by a method modified from sperm ubiquitin assays described previously [34]. Five microliters of Tyrode-Lactate-HEPES medium (TLH) were added per tube to 15 ml Falcon polypropylene centrifuge tubes (Becton Dickinson, Franklin Lakes, NJ), and the thawed contents of each straw were added to the correctly labeled tubes. The use of similar size polystyrene tubes was avoided deliberately as we found previously that many spermatozoa adhere to their walls and are lost during centrifugation. The mixture was spun for 5 minutes at 350×g in a bench-top, fixed angle rotor centrifuge (Centrific; Fischer Scientific) and the supernatant was removed by a Pasteur pipette attached via flexible tube to an air pump, making sure to not disturb the sperm pellet at the bottom of the tube. Next, 5 ml of 2% formaldehyde (EM grade; Polyscience, Warrington, PA) in phosphate buffered saline (PBS) were added to each tube and vortexed. The samples were fixed for 40 min at room temperature (RT) and washed by centrifugation in 8 ml of PBS. After complete removal of the washing buffer, 80 µl of PBS were added per tube. To permeabilize the cells and block potential non-specific antibody binding, 10 µl of 20 % normal goat serum (NGS; Sigma) in PBS with 1 % Triton-100 (Sigma) were added to each tube. After one hour of incubation at room temperature, 10 µl of affinity purified, peptide-specific anti-SPTRX3 antibody [14], diluted1:30 in PBS with 1% NGS and 0.1% TX-100, were added to each tube. The specificity and quantitative nature of this antibody is demonstrated by Western blotting of semen samples from three presumed fertile donors and three patients diagnosed with heritable male infertility (**Figure** **S1 in File S1; Table** **S10 in File S1).** The samples were incubated overnight at 4°C. The next day, 10 µl of goat-anti rabbit IgG conjugated to a red fluorescent dye TRITC (GAR-TRITC; Invitrogen-Zymed; Carlsbad, CA), diluted 1∶9 in PBS with 1% NGS and 0.1% TX-100, were added to each tube. Samples were incubated for 40 min at RT, and washed with 5 ml of PBS-BSA. The washed sperm pellets were resuspended in 500 µl of pure PBS and transferred into 5 ml Falcon flow cytometry tubes (Becton Dickinson, Franklin Lakes, NJ) with cell strainer caps, to remove large debris and agglutinated cells. A shortened version of this protocol was tested on several samples in which fixation and blocking were omitted and the frozen-thawed samples were incubated for 20 min with anti-SPTRX3 antibody diluted in PBS with 1%NGS and 0.1% TX-100. After 20 min, the concentrated GAR-TRITC was added and the samples were viewed under epifluorescence microscope after additional 20 min. of incubation (representative results shown in **Figure** **3H**). None of these samples were included in the flow cytometric analysis.

### Flow Cytometric Analysis

Flow cytometry was performed using FacsScan flow cytometer (Becton Dickinson Corp.), measuring 10,000 cells per sample at 568 nm light wavelength. Acquired relative intensities of TRITC labeling were plotted as histograms and divided into three areas of low (marker area M1), medium (marker area M2) and high relative intensity (marker area M3; see **Figure** **1A, 1B**), essentially as described for ubiquitin, PAFR and 15-LOX proteins in animal spermatozoa [35], [36]. The marker area M1 contains the low-fluorescent sperm fragments and debris. Marker area M2 delimits background fluorescence of intact spermatozoa not carrying SPTRX3, with some content of larger debris particles. Marker area M3 contains the spermatozoa, large sperm-derived debris and immature sperm forms with accumulated, fluorescently labeled SPTRX3 protein (SPTRX3-positive spermatozoa). Blank negative control samples generated by the replacement of anti-SPTRX3 antibody with a non-immune rabbit serum were evaluated in the same trial under identical conditions. For each sample, the following flow cytometric parameters were recorded and entered in MS-Excel tables: Percentage of total cell numbers within marker areas M1-M3 (%M1, %M2, %M3), median fluorescence of all cells in a sample and of the cell subpopulations within marker areas M1-M3 (SPTRX3 Median All, M1, M2, M3), geometric mean of relative fluorescence of all cells in sample (GeoMean), mean relative fluorescence of all cells (Mean All, Mean M1-M3) and cells in M1-M3, coefficient of variation (CV; normalized standard deviation CV  =  SD/Mean), and Peak Channel. Additionally, the % of flow cytometric events registered in visible light scatter as cells of prevailing size out of all events within the whole measured sample were recorded as the % Total values, reflective of relative ratio of combined high and low fluorescence spermatozoa to low-fluorescent small debris in a sperm sample (**see** **Figure** **1A**'**, 1B**').

### Statistical Analysis

Regression analyses were conducted in *SAS* using *proc glm* procedures to investigate the relationship between the SPTRX3 levels and the clinical indication and treatment variables. Logistics regression analysis was conducted using *proc* logistics to investigate the relationship between the pregnancy rate and the SPTRX3 levels, female age variables and treatment assignment. *SAS proc corr* procedure was used to perform a Spearman rank correlation study of relationship between conventional and flow cytometric semen parameters. Discriminant analysis was performed using *SAS proc discrim* and *proc stepdisc* procedures. Cutoff values for diagnosis of male infertility based on %M3 value were established by Receiver Operator Characteristic (ROC) curve analysis. Descriptive statistics and Pearson's correlation coefficients were generated by using statistical tools package in MS-Excel. Values of %M3 and GeoMean were found to be most informative and are listed in the Tables. The value of % TOTAL also showed a strong correlation with some of the clinical semen parameters.

### Analysis by Epifluorescence Microscopy

A randomly chosen subset of 150 samples were examined under a Nikon Eclipse 800 epifluorescence-equipped light microscope to determine the percentage of SPTRX3-positive cells, by using the processed, but unexposed sample remnants left over from flow cytometric measurements. Following the flow cytometric analysis, each leftover sample was transferred from a 5 ml flow cytometry tube to a 1.5 ml Eppendorf tube, sealed and stored at 4°C until mounting and analysis. Filtered PBS was added up to approximately the 0.5 ml mark and 2.5 µl of DNA dye DAPI (200 µg/ml stock) were added to each tube. The samples were spun for 1 minute at 500×g in Sorvall Biofuge Fresco centrifuge to concentrate the sperm in a pellet, of which 7.5 µl were mounted on a clean slide. Slides were covered by coverslips, sealed with clear nail polish and stored at 4°C until evaluation. At least 100 spermatozoa were counted per slide and were categorized into two different groups, SPTRX3-positive cells and SPTRX3-non positive cells. Positive cells were further divided by the association of SPTRX3 labeling with the sperm head or sperm tail. The sperm head category had SPTRX3 in the nuclear vacuoles while the sperm tail category included labeling in the redundant cytoplasm around the proximal midpiece as well as the association of SPTRX3 extending from the sperm tail connecting piece toward the post-acrosomal sheath of the sperm head. The percentages of all SPTRX3-positive spermatozoa and the percentages of head/tail-positive spermatozoa were entered in MS-Excel tables and correlated with flow cytometric data. Representative images were acquired by a CoolSnap HQ CCD camera operated by MetaMorph software, using appropriate epifluorescence filters on a Nikon Eclipse E800 microscope. Images were edited using Adobe® Photoshop CS5 Extended version (Adobe Systems, Inc., San Jose, CA, USA).

## Supporting Information

File S1 **Supplemental figures and tables.** Figure S1. Western blotting of SPTRX3 in semen of three ART patients and three presumed fertile donors (semen parameters are shown in Table S10). Relative levels of SPTRX3 (top panel) and beta-tubulin (loading control; middle panel) are shown for three presumably fertile sperm bank donors with acceptable clinical semen parameters (lanes 1-3) and three teratozoospermic infertile men (lanes 4-6) suffering from dysplasia of the fibrous sheath. Diagram of SPTRX3/tubulin band density ratios is shown on the bottom. Note the high density of SPTRX3 band in donor #1, who was presumed fertile. Figure S2. Smoking has a modest effect on sperm SPTRX3 levels. Heavy smokers consuming 15-30 cigarettes per day had higher average levels of sperm SPTRX3 than other groups shown. Of note, two thirds (10/15) of these heavy smokers had indication/clinical diagnosis of male infertility. The numeric difference between smokers consuming 2-5 cigarettes per day and smokers consuming 15-30 cigarettes per day, was not statistically significant (p>0.1). Figure S3. Scatter diagram illustrating the relationship between subjective, light microscopic assessment of sperm SPTRX3 content (% spermatozoa with SPTRX3-positive heads; x-axis) and flow cytometry (%M3 SPTRX-value; y-axis). This simple light microscopic analysis was conducted as a potential precursor of a test suitable for clinical andrology laboratories. Samples from 150 randomly chosen donors, processed for flow cytometry, were re-evaluated by epifluorescence microscopy for the percentage of SPTRX3 positive sperm heads, sperm tails, and total % of SPTRX3 positive spermatozoa. Correlations were found between all three categories of light microscopic evaluation and % of SPTRX3-positive spermatozoa as measured by flow cytometry (%M3). The highest correlation coefficient (r = 0.46) was between % SPTRX3-positive sperm heads by light microscopy and %M3 SPTRX3 by flow cytometry (see Table S4B). Table S1. Receiver Operator Characteristic (ROC) analysis. As anticipated, Specificity increases at the expense of Sensitivity as the cutoff values increase. The area under the ROC curve is 0.74, which shows that the %M3 performs well for predicting the male infertility. Table S2. Semen SPTRX3 levels and live births. Pregnancy outcomes were available for 57/60 pregnant couples. One of those 57 pregnancies was an ectopic pregnancy in a case of female-only infertility; it is not included in the above table. Live birth was achieved in 81% (46/57) of those analyzed pregnant couples. One of those three male factor patients had a vasectomy. Table S3. Parameters of zygotic development in 239 couples divided into subgroups based on percentages of SPTRX3-positive spermatozoa (A) or clinical indication (B). SPTRX3 values, but not the clinical indication, were predictive of good zygotic development after IVF or ICSI. Couples with lowest SPTRX3 levels (A, top row, <5% M3) produced the highest percentage of normal, two-pronuclear (2 PN) zygotes out of all fertilized oocytes (A, column 8), and also when calculated based on all oocytes harvested (A, column 9). These couples also produced the most embryos suitable for transfer or cryopreservation (column 13). Note that couples with low or medium levels of SPTRX3 produced more 2PN zygotes per couple on average than couples with >15% SPTRX3 (A, column 10). Idiopathic couples in which men recorded more than 15% SPTRX3-positive spermatozoa had the lowest yields of two-pronuclear zygotes. Numbers shown in red are highest & lowest values for each column. No significant correlations were found between parameters of early embryo development and flow cytometric SPTRX3 levels. Table S4. Relationship between the clinical treatment assignment and various sperm quality parameters. Table S5. A: Pregnancy rates in 150 infertile couples, divided by percentages of SPTRX3-positive spermatozoa. In accordance with flow cytometric SPTRX3-data, couples with lowest levels of SPTRX3 had highest pregnancy rate (column 2). Also similar to flow cytometric results, the percentage of couples treated by ICSI increased progressively with sperm SPTRX3 content (column 6). Spermatozoa were evaluated for the presence of SPTRX3 in the sperm head (B) or tail (C; combined head and tail labeling has not been observed), adding up to third category of total SPTRX3-positive spermatozoa (A). Numbers shown in red are highest & lowest values for each column. With the exception of one SPTRX3 parameter (A-% of all positive spermatozoa), pregnancy rates (column #2) in light microscopic analysis were dose-dependent on SPTRX3 values. B: Correlations between light microscopic SPTRX3-evaluation (STIX assay) and % of SPTRX3-positive spermatozoa as measured by flow cytometry. Bold font indicates highest correlation coefficients for flow cytometric and light microscopic parameters. Table S6. Sperm SPTRX3 levels and oocyte quality parameters in 238 couples divided by female partners’ age, with the threshold of 35 years of age. Female age subgroups (in rows) were further divided based on male partners’ semen content of SPTRX3-positive spermatozoa (Column 4), and based on the treatment (IVF or ICSI, columns 6 & 7). Regardless of female partner’s age, the combined IVF & ICSI pregnancy rates (column 9) as well as the ICSI pregnancy rates (column 10) were numerically higher when the male partner had <10% SPTRX3-positive spermatozoa. Table S7. Analysis of subgroups divided by indication (male or female infertility) and female age. Combined and idiopathic cases were not included in analysis. Table S8. Correlations between flow cytometric SPTRX3 values and clinical semen parameters. R-values by Person’s correlation analysis are shown. Most significant values are printed bold. In general, SPTRX3 values correlate negatively with sperm count, motility, and percentage of morphologically normal spermatozoa in samples, assessed by conventional light microscopic semen evaluation. Ratio of spermatozoa to debris (%TOTAL) correlates positively with sperm count, motility and normal morphology. Bold font indicates highest correlation coefficients. Table S9. Spearman correlations between the most informative flow cytometric SPTRX3 parameters and the conventional semen parameters (the second numbers in the cells are the p-values). Table S10. Conventional semen parameters for semen samples used for Western blotting. Table S11. Discriminant analysis using SAS PROC discrim and stepdisc procedures applied to study the relationship of pregnancy rate with the sperm quality parameters and SPTRX3 levels, with consideration of treatment assignments. Table S12. Structure matrix of the discriminant analysis conducted to study the relationship of the treatment assignment, SPTRX3 and the sperm quality parameters. Table S13. Variables and treatment groups in the discriminant analysis conducted to study the relationship of the treatment assignment, SPTRX3 and the sperm quality parameters. Table S14. Canonical structure matrix of the discriminant analysis using SAS proc discrim procedure, conducted to explore the relationships between the clinically diagnosed male infertility and sperm quality parameters. Table S15. Means of the nine variables shown separately for infertile and fertile male in the discriminant analysis (SAS proc discrim) conducted to explore the relationships between the clinically diagnosed male infertility and sperm quality parameters.
(PDF)
