# Correction: Semen Levels of Spermatid-Specific Thioredoxin-3 Correlate with Pregnancy Rates in ART Couples

**DOI:** 10.1371/annotation/5ec7a768-7129-460f-9290-57ae5d631dd9

**Published:** 2013-10-15

**Authors:** Clayton Buckman, Christophe Ozanon, Jing Qiu, Miriam Sutovsky, Joyce Ann Carafa, Vanesa Y. Rawe, Gaurishankar Manandhar, Antonio Miranda-Vizuete, Peter Sutovsky

Affiliation number 7 for the eighth author, Antonio Miranda-Vizuete, is incorrect. 

The correct affiliation is: Instituto de Biomedicina de Sevilla, Hospital Universitario Virgen del Rocío/CSIC/Universidad de Sevilla, Sevilla, Spain. 

