# Supplementary material for: Semen Levels of Spermatid-Specific Thioredoxin-3 Correlate with Pregnancy Rates in ART Couples
Source: PLoS One. 2013 May 29;8(5):e61000. doi: 10.1371/journal.pone.0061000 (PMC3667087; doi:10.1371/journal.pone.0061000)
Supplement: File S1 — Supplemental figures and tables. Figure S1. Western blotting of SPTRX3 in semen of three ART patients and three presumed fertile donors (semen parameters are shown in Table S10). Relative levels of SPTRX3 (top panel) and beta-tubulin (loading control; middle panel) are shown for three presumably fertile sperm bank donors with acceptable clinical semen parameters (lanes 1-3) and three teratozoospermic infertile men (lanes 4-6) suffering from dysplasia of the fibrous sheath. Diagram of SPTRX3/tubulin band density ratios is shown on the bottom. Note the high density of SPTRX3 band in donor #1, who was presumed fertile. Figure S2. Smoking has a modest effect on sperm SPTRX3 levels. Heavy smokers consuming 15-30 cigarettes per day had higher average levels of sperm SPTRX3 than other groups shown. Of note, two thirds (10/15) of these heavy smokers had indication/clinical diagnosis of male infertility. The numeric difference between smokers consuming 2-5 cigarettes per day and smokers consuming 15-30 cigarettes per day, was not statistically significant (p>0.1). Figure S3. Scatter diagram illustrating the relationship between subjective, light microscopic assessment of sperm SPTRX3 content (% spermatozoa with SPTRX3-positive heads; x-axis) and flow cytometry (%M3 SPTRX-value; y-axis). This simple light microscopic analysis was conducted as a potential precursor of a test suitable for clinical andrology laboratories. Samples from 150 randomly chosen donors, processed for flow cytometry, were re-evaluated by epifluorescence microscopy for the percentage of SPTRX3 positive sperm heads, sperm tails, and total % of SPTRX3 positive spermatozoa. Correlations were found between all three categories of light microscopic evaluation and % of SPTRX3-positive spermatozoa as measured by flow cytometry (%M3). The highest correlation coefficient (r = 0.46) was between % SPTRX3-positive sperm heads by light microscopy and %M3 SPTRX3 by flow cytometry (see Table S4B). Table S1. Receiver Ope [file pone.0061000.s001.pdf]

FILE S1 - SUPPLEMENTAL INFORMATION

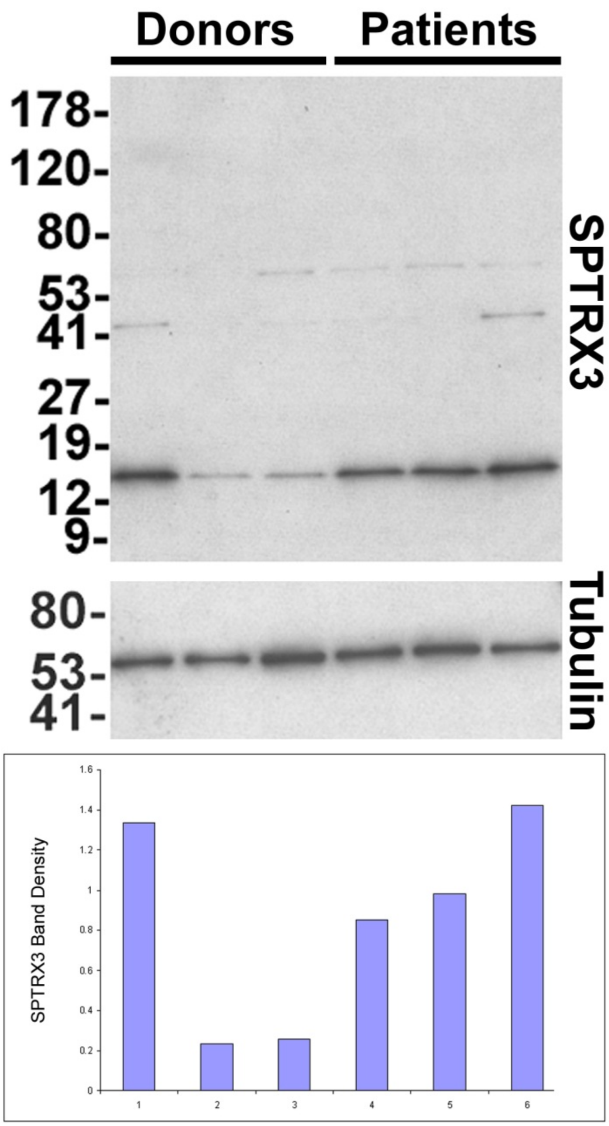

Figure S1: Western blotting of SPTRX3 in semen of three ART patients and three presumed fertile donors (semen parameters are shown in **Table S10**). Relative levels of SPTRX3 (top panel) and beta-tubulin (loading control; middle panel) are shown for three presumably fertile sperm bank donors with acceptable clinical semen parameters (lanes 1-3) and three teratozoospermic infertile men (lanes 4-6) suffering from dysplasia of the fibrous sheath. Diagram of SPTRX3/tubulin band density ratios is shown on the bottom. Note the high density of SPTRX3 band in donor #1, who was presumed fertile.

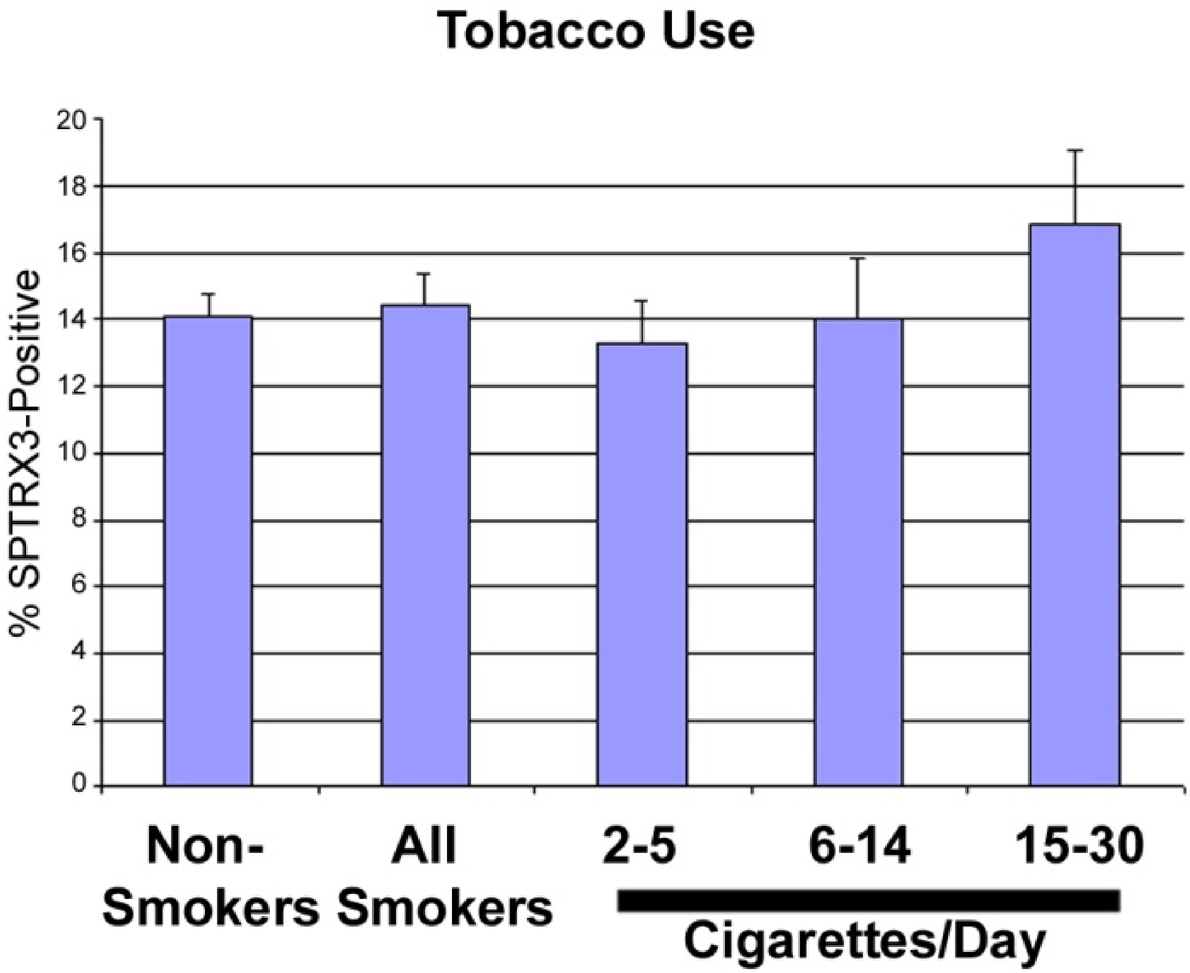

Figure S2: Smoking has a modest effect on sperm SPTRX3 levels. Heavy smokers consuming 15-30 cigarettes per day had higher average levels of sperm SPTRX3 than other groups shown. Of note, two thirds (10/15) of these heavy smokers had indication/clinical diagnosis of male infertility. The numeric difference between smokers consuming 2-5 cigarettes per day and smokers consuming 15-30 cigarettes per day, was not statistically significant (p>0.1).

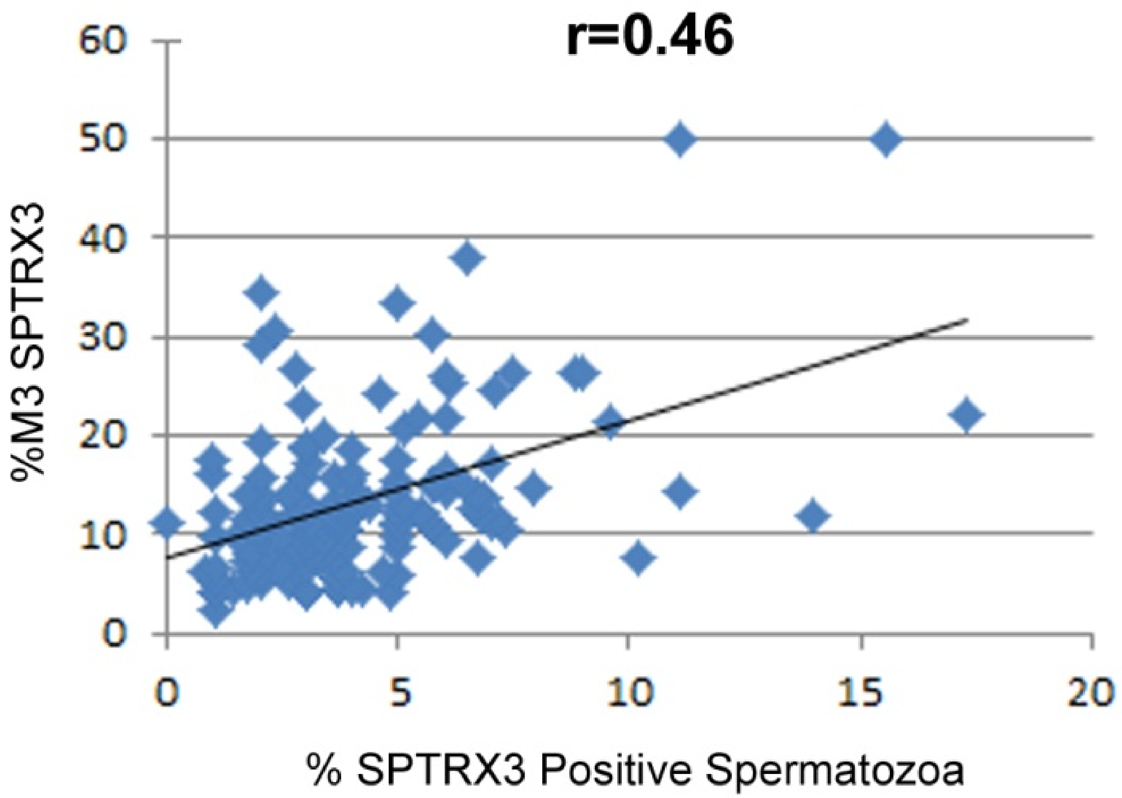

Figure S3: Scatter diagram illustrating the relationship between subjective, light microscopic assessment of sperm SPTRX3 content (% spermatozoa with SPTRX3-positive heads; x-axis) and flow cytometry (%M3 SPTRX-value; y-axis). This simple light microscopic analysis was conducted as a potential precursor of a test suitable for clinical andrology laboratories. Samples from 150 randomly chosen donors, processed for flow cytometry, were re-evaluated by epifluorescence microscopy for the percentage of SPTRX3 positive sperm heads, sperm tails, and total % of SPTRX3 positive spermatozoa. Correlations were found between all three categories of light microscopic evaluation and % of SPTRX3-positive spermatozoa as measured by flow cytometry (%M3). The highest correlation coefficient (r=0.46) was between % SPTRX3-positive sperm heads by light microscopy and %M3 SPTRX3 by flow cytometry (see Supplemental Table 4 B).

Materials & Methods for Western Blotting and Computerized Densitometry

Western blotting was performed to validate the SPTRX3 antibody and to explore the relationship between the total amount of SPTRX3 in spermatozoa of men with good and poor clinical semen parameters. As described previously [36], semen samples were washed, extracted in 50 mM Tris HCl, 500 mM NaCl, 0.5% Triton X-100, 1 mM dithiothreitol, 1 mM phenylmethylsulfonyl fluoride, and a 1:1,000 dilution of protease inhibitor cocktail (chymostatin, leupeptin, antipain, and pepstatin A; Sigma, St. Louis, MO, USA), and boiled for 3 min and loading on a 4-15% gradient tris-glycine gel (Bio-Rad Laboratories, Hercules, CA, USA). Protein load per lane was 30 µg. Five microliters of ProSieve® Color Protein Markers (Cambrex, East Rutherford, NJ, USA) diluted in 5 µl of loading buffer were used as the molecular mass marker. Electrophoresis was performed for one hour using the Fischer Bioscience vertical electrophoresis system. After electrophoresis, proteins were transferred to a polyvinylidene fluoride (PVDF) membrane (Millipore Corp., Bedford, MA, USA) using a wet transfer system (Owl Scientific, Inc., Woburn, MA, USA). The membrane was washed in 0.25% Tris Base Saline (TBS)-Tween 20, five times for 4 min on a platform rocker (Barnstead International, Dubuque, IA, USA). The membrane was then incubated for one hour in 0.25% TBS-Tween 20 and 10% non-fat dried milk for blocking of non-specific antibody binding, washed and incubated overnight with anti-SPTRX3 antibody diluted 1:2,000. On the second day, the membrane was washed and incubated for 1 hr with GAR-IgG HRP (Invitrogen-Zymed) in 0.25% TBS-Tween 20 and 5% non-fat dried milk. The membrane was washed, incubated for 5 min in SuperSignal® West Pico Chemiluminescent Substrate (Pierce Biotechnology, Rockford, IL, USA), and developed on Kodak BioMax Light Film (Kodak, New Haven, CT, USA) for 30 sec using a Hope Micro-Max table-top film processor (Hope, Warminster, PA, USA). The membrane was then stripped and re-probed overnight with mouse monoclonal antibody E7, recognizing beta tubulin (TUBB) (Developmental Studies Hybridoma Bank, University of Iowa, Iowa City, IA), diluted 1:2,000, followed by washing and incubation with goat anti-mouse IgG-HRP, serving as a loading control and densitometric standard. Densitometry was performed by the Kodak Electrophoresis Documentation and Analysis System 290 (EDAS 290) with image capture by the Kodak DC 290 camera. Image analysis was performed by Kodak 1D Image Analysis software (Kodak Scientific Imaging Systems, New Haven, CT, USA) and the relative densities were entered in MS-Excel to calculate the SPTRX3-TUBB ratio for each sample.

TABLE S1: Receiver Operator Characteristic (ROC) analysis. As anticipated, Specificity increases at the expense of Sensitivity as the cutoff values increase. The area under the ROC curve is 0.74, which shows that the %M3 performs well for predicting the male infertility.

Cutoff for %M3	Number of men with %M3 above cutoff	TRUE Positive (TP)	TRUE negative (TN)	False negative (FN)	False Positive (FP)	Sensitivity TP/ (TP+FN)	Specificity TN/ (TN+FP)
**50.0%**	0	0	146	93	0	0.0%	100.0%
**35.0%**	7	6	145	87	1	6.5%	99.3%
**32.5%**	10	9	145	84	1	9.7%	99.3%
**30.0%**	16	13	143	80	3	14.0%	98.0%
**27.5%**	21	17	142	76	4	18.3%	97.3%
**25.0%**	30	23	139	70	7	24.7%	95.2%
**22.5%**	38	30	138	63	8	32.3%	94.5%
**20.0%**	46	34	134	59	12	36.6%	91.8%
**17.5%**	56	39	129	54	17	41.9%	88.4%
**15.0%**	79	49	116	44	30	52.7%	79.5%
**12.5%**	110	63	99	30	47	67.7%	67.8%
**10.0%**	166	80	60	13	86	86.0%	41.1%
**7.5%**	197	88	37	5	109	94.6%	25.3%
**5.0%**	223	90	13	3	133	96.8%	8.9%
**2.5%**	239	93	0	0	146	100.0%	0.0%

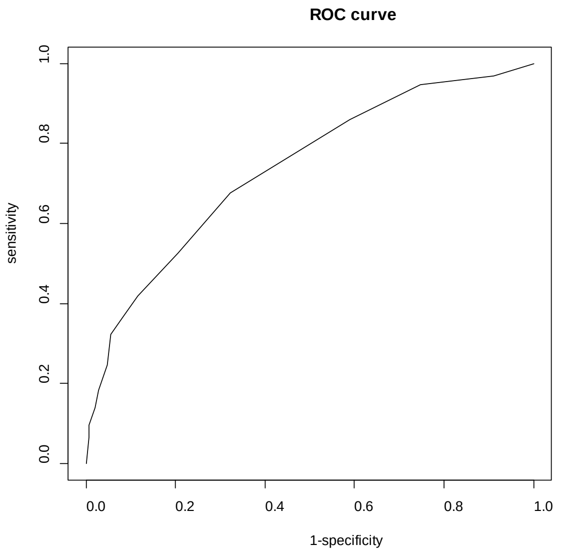

True positive (**TP**) = number of couples ABOVE cutoff value who were diagnosed with male infertility (**MIF**), including couples with combined male & female infertility

True negative (**TN**) = number of couples BELOW cutoff who did NOT have MIF diagnosis

False negative (**FN**) = number of couples BELOW cutoff who were diagnosed with MIF

False positive (**FP)** = number of couples ABOVE cutoff who were NOT diagnosed with MIF

Sensitivity = **TP/(TP+FN)** = percentage of men who were diagnosed with MIF that test positive for the SPTRX**3.**

Specificity = **TN/(TN+FP)** = percentage of men who don't have MIF diagnosis that test negative for the SPTRX3

TABLE S2: Semen SPTRX3 levels and live births.

TABLE S2A:

Pregnancy outcome	**n**	**%M3 SPTRX3**	**Number treated by ICSI (%)**
**Miscarriage – all couples**	10	14.8 +/- 2.2	6 (60%)
**Singleton – all couples**	34	15.3+/-1.6	19 (56%)
**Twins - all couples**	12	10.5 +/- 1.4	7 (58%)
**Miscarriage - male inf. couples**	3	19.8+/-4.0	3 (100%)
**Singleton - male inf. couples**	16	18.2+/-2.7	15 (94%)
**Twins - male inf. couples**	3*	14.5+/-2.5	3 (100%)

Pregnancy outcomes were available for 57/60 pregnant couples. One of those 57 pregnancies was an ectopic pregnancy in a case of female-only infertility; it is not included in the above table. Live birth was achieved in 81% (46/57) of those analyzed pregnant couples.

*One of those three male factor patients had a vasectomy

TABLE S2 B:

**Outcome/% M3 SPTRX3**	**<5%**	**5-9.99%**	**10-14.99%**	**>15%**	**TOTAL**
**Miscarriage – all couples**	0 (0%)	2 (20%)	3 (30%)	5 (50 %)	10
**Singleton – all couples**	3 (9%)	7 (21%)	10 (29%)	14 (41%)	34
**Twins - all couples**	2 (17%)	3 (25%)	5 (41%)	2 (17%)	12
**All live birth couples**	5 (9%)	10 (18%)	15 (26%)	16 (27%)	56
**All pregnant couples**	5 (8%)	13 (22%)	21 (35%)	21 (35%)	60
**All non-pregnant couples**	12 (7%)	44 (25%)	66 (35%)	57 (32%)	179
**ALL couples**	17 (7%)	57 (24%)	87 (35%)	78 (33%)	239

TABLE S3: Parameters of zygotic development in 239 couples divided into subgroups based on percentages of SPTRX3-positive spermatozoa (**A**) or clinical indication (**B**). SPTRX3 values, but not the clinical indication, were predictive of good zygotic development after IVF or ICSI. Couples with lowest SPTRX3 levels (**A**, top row, <5% M3) produced the highest percentage of normal, two-pronuclear (2 PN) zygotes out of all fertilized oocytes (**A,** column 8), and also when calculated based on all oocytes harvested (**A,** column 9). These couples also produced the most embryos suitable for transfer or cryopreservation (column 13). Note that couples with low or medium levels of SPTRX3 produced more 2PN zygotes per couple on average than couples with >15% SPTRX3 (**A,** column 10). Idiopathic couples in which men recorded more than 15% SPTRX3-positive spermatozoa had the lowest yields of two-pronuclear zygotes. Numbers shown in red are highest & lowest values for each column. No significant correlations were found between parameters of early embryo development and flow cytometric SPTRX3 levels.

TABLE S3A: *Parameters of pronuclear development in couples with varied sperm SPTRX3 content*

**Column 1**	**2**	**3**	**4**	**5**	**6**	**7**	**8**	**9**	**10**	**11**	**12**	**13**
**%M3 SPTRX3**	**n - couples**	**Total oocytes matured**	**Total oocytes fertilized**	**Total polypronuclear zygotes (%)**	**Total 0 PN zygotes (%)**	**Total 1 PN zygotes (%)**	**Total 2 PN zygotes produced (% of fertilized oocytes)**	**% 2 PN zygotes produced out of all harvested oocytes**	**Avg. number of 2PN zygotes produced per couple**	**Embryos transferred (% out of all 2 PN zygotes)**	**Embryos cryo-preserved (out of all 2 PN zyg)**	**% Embryos transferred and cryo-preserved out of all 2 PN zygotes**
<5%	16	167	159	9	9	4	137 (**86.2%)**	**82.0%**	8.6	30 (21.9%)	84 (61.3%)	83.2%
5-9.99%	57	732	665	45	78	30	512 (77.0%)	69.9%	**9.0**	123 (24.0%)	268 (52.3%)	76.3%
10-14.99%	88	995	793	36	103	39	615 (77.6%)	61.8%	7.0	174 (28.3%)	319 (52.3%)	80.6%
>15%	78	871	679	25	65	26	563 (82.9%)	64.6%	7.2	164 (29.1%)	258 (45.8%)	74.9%
>15% idiopathic	12	148	109	6	25	2	76 **(69.7%)**	**51.4%**	**6.3**	25(16.9%)	38 (50.0%)	66.9%

TABLE S3B: *Lack of influence of clinical indication on parameters of pronuclear development*

Male Inf.	72	818	654	25	34	23	572 (87.5%)	69.9%	7.9	162 (28.3%)	301 (52.6%)	80.9%
Female Inf.	85	963	899	59	131	51	658 (73.2%)	68.3%	7.7	168 (25.5%)	342 (52.0%)	77.5%
Combined Inf.	21	283	220	3	12	4	201 (91.4%)	71.0%	9.6	39 (19.4%)	98 (48.8%)	68.2%
Idiopathic Inf.	61	728	584	28	83	21	452 (77.4)	62.1%	7.4	127 (28.1%)	220 (48.7%)	76.8%

TABLE S4: Relationship between the clinical treatment assignment and various sperm quality parameters.

Parameter	p-value	Odds ratio*
V	0.8554	NA
PR	<0.0001	1.176
NX	<0.0001	1.067
NEC	<0.0001	0.936
M	<0.0001	1.142
C	<0.0001	1.029
Median ALL	0.0125	0.964
Median M3	<0.0001	0.978
Median M2	0.3114	NA
Median M1	0.0022	5.187
Mean all	<0.0001	0.960
Mean M3	<0.0001	0.972
Mean M2	0.3920	NA
Mean M1	0.0001	34.058
Total	<0.0001	1.088
%M3	<0.0001	0.904
%M2	<0.0001	1.141
%M1	0.4735	NA
CVALL	0.0014	0.994
CVM3	0.0357	0.99
CVM2	0.0342	0.931
CVM1	0.0006	0.837

<u>Abbreviations:</u>

C=sperm count

V=semen volume

M=total motility

PR=progressive motility

NX=morphology/ % normal spermatozoa

NEC=percent of necrotic spermatozoa assessed by Williams test

TABLE S5 A: Pregnancy rates in 150 infertile couples, divided by percentages of SPTRX3-positive spermatozoa. In accordance with flow cytometric SPTRX3-data, couples with lowest levels of SPTRX3 had highest pregnancy rate (column 2). Also similar to flow cytometric results, the percentage of couples treated by ICSI increased progressively with sperm SPTRX3 content (column 6). Spermatozoa were evaluated for the presence of SPTRX3 in the sperm head (**B**) or tail (**C**; combined head and tail labeling has not been observed), adding up to third category of total SPTRX3-positive spermatozoa (**A**). Numbers shown in red are highest & lowest values for each column. With the exception of one SPTRX3 parameter (A-% of all positive spermatozoa), pregnancy rates (column #2) in light microscopic analysis were dose-dependent on SPTRX3 values.

**Column #**	**1**	**2**	**3**	**4**	**5**	**6**
**Parameter**	**N (couples)**	**% pregnant (n)**	**% male & combined infertility cases in this group (n)**	**Avg. %2 PN zygotes per couple out of all ovulated (n/total)**	**Avg. %2 PN zygotes per couple out of all fertilized**	**% treated by ICSI**
**A-% of all positive spermatozoa (head-positive and tail-positive, combined)**						
**<5%**	30	**37% (11)**	13% (4)	59.5% (235/395)	**79.1%** (235/297)	**267%** (8/30)
**5-9.99%**	73	29% (23)	19% (14)	49.3% (500/1014)	74.9% (500/668)	48% (35/73)
**10-14.99%**	29	**18% (5)**	38% (11)	59.2% (252/426)	83.7% (252/301)	59% (17/29)
**>15%**	18	22% (4)	56% (10)	58.7% (125/213)	**89.9%** (125/139)	**72%** (13/18)
**All couples**	150	29% (43)	26% (39)	54.3% (1112/2048)	79.2% (1112/1405)	49% (73/150)
**B-% of head only-positive spermatozoa**						
**<5%**	103	33% (34)	19%(20)	53.6% (764/1417)	75.2% (764/1016)	44% (44/103)
**5-9.99%**	41	20% (8)	39% (16)	52.8% (299/566)	88.7% (299/337)	63% (26/41)
**>10%**	6	17% (1)	50% (3)	75.5% (49/65)	94.2% (49/52)	50 % (3/6)
**C-% of tail only-positive spermatozoa**						
**<5%**	93	33% (31)	16% (15)	51.3% (667/1301)	75.9% (667/879)	38% (35/93)
**5-9.99%**	46	22%(10)	39% (18)	59.7% (382/640)	84.0% (382/455)	70% (32/46)
**>10%**	10	20% (2)	60% (6)	58.9% (63/107)	88.7% (63/71)	60% (6/10)

TABLE S5 B: Correlations between light microscopic SPTRX3-evaluation (STIX assay) and % of SPTRX3-positive spermatozoa as measured by flow cytometry. Bold font indicates highest correlation coefficients for flow cytometric and light microscopic parameters.

	% Tail	%Head	%H&T	%M1	%M2	%M3
% Tail	1					
%Head	0.71	1				
%H&T	0.94	0.91	1			
%M1	-0.29	-0.27	-0.31	1		
%M2	-0.25	-0.41	-0.35	0.08	1	
%M3	0.33	**0.46**	**0.42**	-0.41	-0.94	1

Abbreviations:

% Tail=spermatozoa with SPTRX3 labeling in the sperm tail midpiece

%Head: spermatozoa with SPTRX3-labeling in the sperm head (mostly nuclear vacuoles)

%H&T=Combined % of spermatozoa carrying SPTRX3 in either sperm head or tail

%M1=% spermatozoa gated within marker M1 in flow cytometric histograms

%M2=% spermatozoa gated within marker M2 in flow cytometric histograms

%M3=% spermatozoa gated within marker M3 in flow cytometric histograms

Table S6: Sperm SPTRX3 levels and oocyte quality parameters in 238 couples divided by female partners' age, with the threshold of 35 years of age. Female age subgroups (in rows) were further divided based on male partners' semen content of SPTRX3-positive spermatozoa (Column 4), and based on the treatment (IVF or ICSI, columns 6 & 7). Regardless of female partner's age, the combined IVF & ICSI pregnancy rates (column 9) as well as the ICSI pregnancy rates (column 10) were numerically higher when the male partner had <10% SPTRX3-positive spermatozoa.

**1**	**2**	**3**	**4**	**5**	**6**	**7**	**8**	**9**	**10**
Female age	*Avg. male age*	*Avg. % M3 SPTRX3*	*Threshold % M3 SPTRX*	*n*	*Treated by IVF*	*Treated by ICSI (%)*	*Mature oocytes total (per couple)*	*Pregnant couples IVF & ICSI (%)*	*Pregnant ICSI couples*
35 years or younger<br><br>*n=140*	33.2±0.36	14.3±0.75	<10	48	30**	21 (43%)	693 (14.1)	18/48 (38%)	9/21 (43%)
			>10	93	35***	60 (65%)	1193 (12.8)	23/93 (25%)	18/60 (30%)
Older than 35 n=99	40.7±0.6	14.4±0.83	<10	25	20	5 (20%)	206 (8.24)	6/25 (24%)	2/5 (40%)
			>10	74	30****	45 (61%)	673 (9.1)	13/74 (18%)	7/45 (16%)

*Age information was not provided for one female patient
**Three couples were first treated by IVF before ICSI
***Three couples were first treated by IVF before ICSI
****One couple was first treated by IVF before ICSI

TABLE S7: Analysis of subgroups divided by indication (male or female infertility) and female age. Combined and idiopathic cases were not included in analysis.

A. Female infertility couples

Age female	Avg. % M3	Avg. Male age	% M3	n	Treated by IVF	Treated by ICSI (%)	Mature oocytes total (per couple)	Pregnant couples IVF & ICSI (%)	Pregnant ICSI couples
**35 yr or less**	10.5±0.8	41.0 ±1.0	**<10**	24	23	1 (4.3%)	326 (13.6)	7 (30.4%)	0 (0%)
			**>10**	22	17	5 (22.7%)	278 (12.6)	7 (31.8%)	2 (40%)
**>35 yr**	12.5±1.2	32.8±0.6	**<10**	12	11	1 (8.3%)	93 (7.8)	4 (33.3%)	1 (100%)
			**>10**	27	19	8 (29.6%)	213 (7.9)	10 (37.0%)	1 (12.5%)

B. Male infertility couples

Age female	Avg. % M3	Avg. Male age	% M3	n	Treated by IVF	Treated by ICSI (%)	Mature oocytes total (per couple)	Pregnant couples IVF & ICSI (%)	Pregnant ICSI couples
**35 yr or less**	18.3±1.6	33.4±0.7	**<10**	9	0*	9 (100%)	131 (14.6)	2 (22.2%)	2 (22.2%)
			**>10**	37	0**	37 (100%)	448 (12.1)	4 (10.8%)	4 (10.8%)
**>35 yr**	19.4±1.7	41.0±1.0	**<10**	0	0	0 (0%)	0 (0)	0 (0%)	0 (0%)
			**>10**	26	2	24 (92.3%)	239 (9.2)	8 (30.8%)	7 (29.2%)

*Two couples were first treated by IVF before ICSI
**One couple was first treated by IVF before ICSI

TABLE S8: Correlations between flow cytometric SPTRX3 values and clinical semen parameters. R-values by Person's correlation analysis are shown. Most significant values are printed bold. In general, SPTRX3 values correlate negatively with sperm count, motility, and percentage of morphologically normal spermatozoa in samples, assessed by conventional light microscopic semen evaluation. Ratio of spermatozoa to debris (%TOTAL) correlates positively with sperm count, motility and normal morphology. Bold font indicates highest correlation coefficients.

**Sorted by**		**Clinical Parameter**	**Correlation with %M3**	**Correlation with %TOTAL**	**Correlation with GeoMean M3**
**A-None**	All patients n=239	Sperm count	**-0.37**	**0.58**	**-0.55**
		% live sperm.	0.30	-0.19	0.23
		% prog. motile sperm.	**-0.42**	**0.34**	**-0.42**
		% total motile sperm.	**-0.43**	**0.45**	**-0.46**
		% normal sperm.	**-0.35**	0.30	-0.33
**B-Indication:**	Male infertility n=72	Sperm count	-0.24	**0.38**	0.28
		% live sperm.	-0.09	-0.30	-0.26
		% prog. motile sperm.	0.19	0.29	0.20
		% total motile sperm.	0.18	0.24	0.09
		% normal sperm.	-0.04	0.20	-0.06
	Female infertility n=85	Sperm count	-0.17	**0.46**	**-0.38**
		% live sperm.	0.08	-0.07	-0.02
		% prog. motile sperm.	-0.15	0.19	-0.19
		% total motile sperm.	-0.14	0.24	-0.19
		% normal sperm.	-0.17	0.32	-0.21
	Combined infertility n=61	Sperm count	0.02	**0.80**	**-0.46**
		% live sperm.	0.04	-0.35	**0.45**
		% prog. motile sperm.	0.01	**0.39**	**-0.42**
		% total motile sperm.	-0.17	**0.39**	**-0.63**
		% normal sperm.	-0.21	0.02	-0.13
	Idiopathic infertility n=21	Sperm count	**0.35**	0.19	-0.10
		% live sperm.	-0.07	-0.18	-0.11
		% prog. motile sperm.	-0.02	0.25	0.27
		% total motile sperm.	-0.05	0.27	**0.34**
		% normal sperm.	0.23	0.04	-0.04
**C-Pregnancy**	Pregnant n=60	Sperm count	-0.31	0.25	-0.22
		% live sperm.	0.09	-0.24	0.27
		% prog. motile sperm.	-0.30	**0.48**	**-0.44**
		% total motile sperm.	-0.32	**0.46**	**-0.42**
		% normal sperm.	-0.29	0.33	-0.29
	Non-pregnant n=179	Sperm count	-0.32	0.09	-0.06
		% live sperm.	0.31	-0.23	0.22
		% prog. motile sperm.	**-0.36**	0.26	-0.23
		% total motile sperm.	**-0.38**	0.30	-0.26
		% normal sperm.	-0.32	0.19	-0.10
**D-Pregnancy and clinical indication**	Female-pregnant	Sperm count	-0.26	**0.64**	**-0.53**
		% live sperm.	-0.06	-0.15	0.08
		% prog. motile sperm.	-0.24	0.25	-0.31
		% total motile sperm.	-0.28	0.26	**-0.40**
		% normal sperm.	-0.29	**0.55**	**-0.48**
	Female non-pregnant	Sperm count	-0.13	0.40	-0.34
		% live sperm.	0.14	-0.04	-0.07
		% prog. motile sperm.	-0.08	0.15	-0.12
		% total motile sperm.	-0.01	0.21	-0.06
		% normal sperm.	-0.09	0.22	-0.11

	Combined pregnant	Sperm count	**0.64**	**0.72**	**-0.52**
		% live sperm.	-0.22	**-0.37**	0.22
		% prog. motile sperm.	0.05	0.18	**-0.92**
		% total motile sperm.	0.22	**0.36**	**-0.87**
		% normal sperm.	**-0.37**	-0.26	-0.22
	Combined non-pregnant	Sperm count	-0.13	**0.85**	**-0.54**
		% live sperm.	0.12	**-0.50**	**0.59**
		% prog. motile sperm.	0.01	**0.60**	**-0.39**
		% total motile sperm.	-0.28	**0.55**	**-0.60**
		% normal sperm.	-0.21	0.18	-0.14
	Male-pregnant	Sperm count	-0.32	**0.45**	**-0.37**
		% live sperm.	0.21	**0.45**	0.01
		% prog. motile sperm.	-0.27	-0.28	0.05
		% total motile sperm.	-0.24	-0.23	0.16
		% normal sperm.	-0.22	-0.30	0.20
	Male-non-pregnant	Sperm count	-0.24	**0.47**	**-0.42**
		% live sperm.	0.32	-0.26	0.08
		% prog. motile sperm.	-0.35	0.02	-0.21
		% total motile sperm.	-0.27	0.16	-0.08
		% normal sperm.	-0.14	0.06	-0.14
	Idiopathic-pregnant	Sperm count	**-0.50**	0.20	**-0.67**
		% live sperm.	0.04	0.14	**-0.45**
		% prog. motile sperm.	**-0.63**	0.22	-0.27
		% total motile sperm.	**-0.57**	0.26	-0.32
		% normal sperm.	**-0.57**	0.12	-0.15
	Idiopathic-non-pregnant	Sperm count	-0.29	**0.50**	**-0.52**
		% live sperm.	0.26	-0.05	0.24
		% prog. motile sperm.	-0.25	-0.02	-0.08
		% total motile sperm.	-0.25	0.20	-0.27
		% normal sperm.	-0.15	0.01	-0.04
**E-Treatment**	IVF n=98	Sperm count	-0.20	**0.46**	**-0.34**
		% live sperm.	0.04	-0.03	-0.04
		% prog. motile sperm.	-0.14	0.04	-0.05
		% total motile sperm.	-0.14	0.21	-0.08
		% normal sperm.	-0.17	0.16	-0.12
	ICSI n=131	Sperm count	-0.32	**0.51**	**-0.52**
		% live sperm.	0.31	-0.10	0.19
		% prog. motile sperm.	**-0.36**	0.23	**-0.34**
		% total motile sperm.	**-0.37**	0.33	**-0.37**
		% normal sperm.	-0.28	0.14	-0.20

TABLE S9: Spearman correlations between the most informative flow cytometric SPTRX3 parameters and the conventional semen parameters (the second numbers in the cells are the p-values).

	**M**	**PR**	**NEC**	**NX**	**C**
**MeanM1**	0.40<br><0.0001	0.39<br><0.0001	-0.24<br><0.0001	0.27<br><0.0001	0.32<br><0.0001
**MedianM1**	0.37<br><0.0001	0.36<br><0.0001	-0.23<br>0.0004	0.24<br>0.0002	0.26<br><0.0001
**CVM1**	-0.35<br><0.0001	-0.35<br><0.0001	0.24<br><0.0001	-0.23<br>0.0002	-0.28<br><0.0001
**%M2**	0. 50<br><0.0001	0.48<br><0.0001	-0.34<br><0.0001	0.36<br><0.0001	0.48<br><0.0001
**%M3**	-0.45<br><0.0001	-0.43<br><0.0001	0.31<br><0.0001	-0.32<br><0.0001	-0.41<br><0.0001

Abbreviations:

C=sperm count

V=semen volume

M=total motility

PR=progressive motility

NX=morphology/ % normal spermatozoa

NEC=percent of necrotic spermatozoa assessed by Williams test

TABLE S10: Conventional semen parameters for semen samples used for Western blotting.

	PRESUMED FERTILE DONORS			DIAGNOSED, TERATOSPERMIC MALE INFERTILITY PATIENTS		
**SAMPLE NUMBER**	**1**	**2**	**3**	**4**	**5**	**6**
**SPERM COUNT ($x10^6$)**	117	143	205	18	54	44
**% MOTILE**	60	70	65	0.2	0.9	20
**% MORPHOL. DEFECTS**	28	22	26	97	98	87
**AGE**	20	27	23	26	33	34
**SPTRX3/TUBB RATIO**	1.34	0.26	0.23	0.85	0.98	1.42

Supplemental Statistical Information
(Discriminant Function Analysis)

ANALYSIS I: ***Discriminant analysis using SAS PROC discrim and stepdisc procedures was applied to study the relationship of pregnancy rate with the sperm quality parameters and SPTRX3 levels, with consideration of treatment assignments.***

SAS proc discrim procedure was applied to conduct discriminant analysis to understand the relationship between the pregnancy rate and all the sperm quality parameters. When all data is considered and all sperm quality parameters are considered, there is no significant discriminant function. The stepwise discriminant analysis identifies one significant predictor, PR, which leads to a discriminant function with canonical correlation of 0.13 (p-value=0.05). The cross-validated classification accuracy rate based on this discriminant function is 75%. The PR variable separates the two groups well with mean value of 21.6 for the non-pregnant group and 24.8 for the pregnant group.

When considering the IVF group only, no significant discriminant function is detected when all sperm quality parameters are considered. The stepwise discriminant analysis identifies Volume and M2% as significant predictors and the discriminant function based on these two variables is significant with p-value=0.008 and has canonical correlation of 0.3. The discriminant function is highly positively correlated with M2% (r=0.71) and negatively correlated with Volume (r=-0.68). The cross-validated classification accuracy rate of this discriminant function is 73%. The means of M2% are 81.4 versus 84.2 for non-pregnancy group and pregnant group, respectively. The volume has mean 3.4 for non-pregnancy group and 2.8 for pregnancy group.

When considering the ICSI treatment group only, the *stepdisc* procedure identified four predictors: V, PR, M and C. The discriminant function based on these four predictors has p-value=0.014 and canonical correlation of 0.31. The cross-validated classification accurate rate is 78%. The structure matrix shows that this discriminant function is positively correlated with C (r=0.52) and negatively correlated with V (r=-0.5). The C variable separates the two groups well with 44.9 (non-pregnancy group) versus 26.7 (pregnancy group). The V variable has mean of 3.1 (no pregnancy) versus 3.6 (pregnancy).

If only the biomarker parameters are considered, the discriminant analysis identified %M2 and CVM3 as significant predictors for the IVF group (**Table S11**). The discriminant function has p-value 0.025 with canonical correlation of 0.26. It has positive correlation with %M2 (r=0.82) and CVM3 (r=0.77). The cross validated classification accuracy rate is 72% and the two variables %M2 and CVM3 separates the pregnancy group from the no-pregnancy group well.

If only the biomarker parameters are considered, the discriminant analysis identified Median M3 as a significant predictor for the ISCI group. The discriminant function has p-value 0.05 with canonical correlation of 0.17. The cross validated classification accuracy rate is 80% and the variable Median M3 separates the pregnancy group from the no-pregnancy group well with means of 232 versus 212 for the two groups.

TABLE S11:

**Pregnancy**	**Obs**	**Variable**	**N**	**Mean**	**Std Dev**	**Minimum**	**Maximum**
YES	75	%M2	75	81.43	6.33	47.92	89.43
		CVM3	75	156.85	27.76	89.42	230.44
NO	32	%M2	32	84.18	4.03	72.95	89.42
		CVM3	32	169.81	31.11	98.96	258.01

ANALYSIS II: ***Discriminant analysis was conducted to study the relationship of the treatment assignment, SPTRX3 and the sperm quality parameters. SAS proc discrim procedure was used to conduct discriminant analysis to study the relationship between the treatment assignment and the sperm quality parameters.***

The discriminant function is highly significant (p<0.0001) with canonical correlation of 0.67. The structure matrix in **TABLE S12** shows that the discriminant function is positively correlated with PR (0.78), C (0.62), M (0.59) NX (0.54), % Total (0.48), %M2% (0.43) and negatively correlated with Mean M3 (-0.5), Median M3 (-0.43), NX (% normal sperm) (-0.39) and %M3 (-0.37). The cross-validated classification accuracy rate based on this discriminant function is 80%. Note that the above 10 predictors include the same nine predictors in the analysis of male infertility indicators besides the NX variable.

TABLE S12: Pooled Within Canonical Structure

Variable	***Can1***
M1	-0.041339
M2	0.431106
M3	-0.369862
Total	0.478822
Meanall	-0.259177
MeanM1	0.294226
MeanM2	-0.067757
MeanM3	-0.503966
CVall	-0.226949
CVM1	-0.251488
CVM2	-0.143186
CVM3	-0.134124
MedianALL	-0.197052
MedianM1	0.226282
MedianM2	-0.080284
MedianM3	-0.432187
V	0.023715
C	0.616992
NEC	-0.390293
M	0.588741
PR	0.779281
NX	0.544063

Abbreviations: C=sperm count; V=semen volume; M=total motility; PR=progressive motility; NX=morphology/ % normal spermatozoa; NEC=percent of necrotic spermatozoa assessed by Williams test

TABLE S13 gives the means of the ten variables by the treatment groups and shows that the ten variables separate the two groups well. For instance, %M2 has a mean of 82.25 for the IVF group and a mean of 76.41 for the ICSI group.

Table S13:

Treatment	***Obs***	***Variable***	***Mean***	***N***
**IVF**	107	%M2	82.2496262	107
		%M3	11.3400935	107
		Total	89.8795327	107
		MeanM3	229.6221495	107
		MedianM3	184.2907477	107
		C	95.7102804	107
		NX	24.4439252	107
		M	47.5981308	107
		PR	29.2523364	107
		NX	49.8773585	106
**ICSI**	131	%M2	76.4079389	131
		%M3	16.8411450	131
		Total	80.2452672	131
		MeanM3	274.9711450	131
		MedianM3	215.6846565	131
		C	41.1854962	131
		NX	32.1267717	127
		M	34.6564885	131
		PR	16.5590551	127
		NX	34.1111111	126

Abbreviations: C=sperm count; V=semen volume; M=total motility; PR=progressive motility; NX=morphology/ % normal spermatozoa; NEC=percent of necrotic spermatozoa assessed by Williams test

ANALYSIS III: ***Logistic regression analysis using SAS proclogistic procedure was applied to study the relationship of pregnancy rate with the sperm quality parameters with consideration of female age and treatment assignments.***

Considering only the patients assigned to the IVF treatment, both %M2 SPTRX3 and %M3 SPTRX3 were significant predictors for pregnancy rate, with or without adjustment for the female age factor. None of the convention parameters or other flow cytometric parameters was significant even with adjustment for the female age. The odds of getting pregnant in the IVF group were reduced by 10.2% for every unit of increase in %M3 (p-value=0.03). After adjusting for the factor of female partner's age (35 years or younger vs. older than 35), the odds of getting pregnant in the IVF group were reduced by 11.1% for every unit of increase in %M3 (p-value=0.03). The odds of getting pregnant when %M3 was below 5% were 6.7 times better than for the %M3 levels above 15% (p-value=0.05). After adjusting for female age, the adjusted odds ratio was 7.5 with p-value=0.04. We found that %M2 was also a significant predictor for pregnancy rate for IVF group with p-value=0.03 and the odds ratio is 1.1 for each unit increase. After adjusting for the female age, the p-value=0.02 and the odds ratio was 1.1 (i.e. the odds of getting pregnant in IVF group increased by 13.6% for every unit increase of %M2).

For the ICSI group, the pregnancy rate was not affected by any of the new or conventional sperm quality parameters or the female age factors. When considering all patients together, %M3 and %M2 were not significant predictors for pregnancy rate (p-value=0.38 and 0.50 respectively). However, when we considered categorizing the %M3 levels into four groups, below 5%, between 5-10%, between 10-15%, and above 15% SPTRX3-positive spermatozoa, the estimated odds ratio between the below 5% group and the above 15% group was 2.94 with p-value=0.06. In other words, the odds of getting pregnant when the SPTX3 levels was below 5% were 2.94 times better than that for the SPTX3 levels above 15%. After adjusting for female age, the adjusted odds ratio for the below 5% SPTX3 group over the above15% SPTX3 group was 3.3 with p-value=0.04. Using 85% as a cutoff for %M2, the below 85% group and above 85% group had significantly different pregnancy rate when considering all patients together. The odds of getting pregnant for the above 85% of %M2 group were 113.2% better than that for the below 85% group (p-value=0.02) and the pregnancy rate was 41.5% versus 22.72%. After the adjustment for the female age, the odds ratio was 2.1 with p-value=0.02.

ANALYSIS IV: ***Discriminant analysis using SAS proc discrim procedure was conducted to explore the relationships between the clinically diagnosed male infertility and sperm quality parameters. Both male-only and combined (female & male) infertility cases are considered to be male infertile cases in this analysis of various sperm quality measurements including the conventional parameters and the parameters based on SPTRX3 analysis by flow cytometry.***

SAS proc discrim procedure was used to study the relationship between the male infertility indicator variable and all the sperm quality parameters. The discriminant function detected is highly significant with p-value<0.0001 and has the canonical correlation of 0.83, which is the maximal multiple correlation of the linear combination of the sperm quality parameters with the male infertility indicator variable. **TABLE S14** (below) presents the canonical structure matrix of the discriminant analysis, which shows that the discriminant function is positively correlated with progressive motility (PR; r=0.84), total motility (M; r=0.60) and % normal spermatozoa (NX; r=0.46), C (r=0.46), % Total (r=0.35), %M2 (r=0.33) and negatively correlated with %M3 (r=-0.29) Mean M3(r=-0.35) and Median M3(r=-0.30). The cross-validated classification accuracy rate based on the discriminant function is 90%. **TABLE S15** shows means of the above nine variables separately for infertile and fertile male. Based on Table 13, all nine variables (PR, M, NX, C, % Total, % M2, % M%, Mean M3 and Median M3) clearly separate the two groups. For example, PR separates the two groups with a mean of 11.5 for infertile male and 29 for fertile male. %M3 has mean 18.6 for infertile group versus mean 11.6 for the fertile group, which is in accordance with the proposed property of SPTRX3 protein as a biomarker specific to defective spermatozoa associated with male infertility.

TABLE S14: Canonical structure matrix of the discriminant analysis of the relationship between male infertility and sperm quality.

Pooled Within Canonical Structure

Variable	*Can1*
M1	0.005240
M2	0.327101
M3	-0.294292
Total	0.345735
MeanAll	-0.231519
MeanM1	0.215277

MeanM2	-0.118957
MeanM3	-0.349959
CVall	-0.151343
CVM1	-0.186697
CVM2	-0.024542
CVM3	-0.100571
MedianALL	-0.192479
MedianM1	0.210773
MedianM2	-0.126937
MedianM3	-0.299451
V	0.021139
C	0.456325
NEC	-0.244176
M	0.601372
PR	0.841907
NX	0.458204

TABLE S15: Means of the nine variables for infertile male and fertile male

Mean	***Infertile male***	***Fertile male***
PR	11.5	29
M	29.3	47.6
NX	28.6	49.1
C	27.2	90.3
% Total	77.8	88.9
M2% SPTRX3	78.8	81.8
M3% SPTRX3	18.6	11.6
MeanM3 SPTRX3	285.5	234.7
MedianM3 SPTRX3	223.1	187.7
Abbreviations: C=sperm count; V=semen volume; M=total motility; PR=progressive motility; NX=morphology/% normal spermatozoa		
